# Methylation differences in Alzheimer’s disease neuropathologic change in the aged human brain

**DOI:** 10.1186/s40478-022-01470-0

**Published:** 2022-11-29

**Authors:** Anna-Lena Lang, Tiffany Eulalio, Eddie Fox, Koya Yakabi, Syed A. Bukhari, Claudia H. Kawas, Maria M. Corrada, Stephen B. Montgomery, Frank L. Heppner, David Capper, Daniel Nachun, Thomas J. Montine

**Affiliations:** 1 Department of Neuropathology, Charité–Universitätsmedizin Berlin, Corporate member of Freie Universität Berlin and Humboldt-Universität Zu Berlin, Charitéplatz 1, 10117 Berlin, Germany; 2 Department of Pathology, Stanford University, 300 Pasteur Drive, Stanford, CA 94305 USA; 3 Department of Genetics, Stanford University, Stanford, CA 94305 USA; 4 Department of Biomedical Data Science, Stanford University, Stanford, CA 94305 USA; 5 Department of Neurology, University of California Irvine, Orange, CA 92868-4280 USA; 6 Department of Epidemiology, University of California, Irvine, CA 92617 USA; 7 Department of Neurobiology and Behavior, University of California, Irvine, CA 92697 USA; 8 German Center for Neurodegenerative Diseases (DZNE), 10117 Berlin, Germany; 9 Cluster of Excellence, NeuroCure, 10117 Berlin, Germany

**Keywords:** Methylation, Brain, Deconvolution, 90 plus, Old age, Alzheimer’s disease, AD, PEN-2, Gamma secretase

## Abstract

Alzheimer’s disease (AD) is the most common cause of dementia with advancing age as its strongest risk factor. AD neuropathologic change (ADNC) is known to be associated with numerous DNA methylation changes in the human brain, but the oldest old (> 90 years) have so far been underrepresented in epigenetic studies of ADNC. Our study participants were individuals aged over 90 years (n = 47) from *The 90*+ *Study*. We analyzed DNA methylation from bulk samples in eight precisely dissected regions of the human brain: middle frontal gyrus, cingulate gyrus, entorhinal cortex, dentate gyrus, CA1, substantia nigra, locus coeruleus and cerebellar cortex. We deconvolved our bulk data into cell-type-specific (CTS) signals using computational methods. CTS methylation differences were analyzed across different levels of ADNC. The highest amount of ADNC related methylation differences was found in the dentate gyrus, a region that has so far been underrepresented in large scale multi-omic studies. In neurons of the dentate gyrus, DNA methylation significantly differed with increased burden of amyloid beta (Aβ) plaques at 5897 promoter regions of protein-coding genes. Amongst these, higher Aβ plaque burden was associated with promoter hypomethylation of the Presenilin enhancer 2 (*PEN-2*) gene, one of the rate limiting genes in the formation of gamma-secretase, a multicomponent complex that is responsible in part for the endoproteolytic cleavage of amyloid precursor protein into Aβ peptides. In addition to novel ADNC related DNA methylation changes, we present the most detailed array-based methylation survey of the old aged human brain to date. Our open-sourced dataset can serve as a brain region reference panel for future studies and help advance research in aging and neurodegenerative diseases.

**Supplementary Information:**

The online version contains supplementary material available at 10.1186/s40478-022-01470-0.

## Introduction

Alzheimer’s disease (AD) is the most common form of dementia. AD Neuropathological changes (ADNC) include extracellular aggregates of the amyloid beta (Aβ) peptides into plaques, intraneuronal formation of hyperphosphorylated paired helical filament (PHF) tau into neurofibrillary tangles, and neuritic plaques that are composed of both extracellular Aβ and PHF tau in neuronal processes. Moreover, neuroinflammation mainly characterized by an activation of glial cells is known to be yet another pathogenetic component of AD. In cohorts with mean age of 78–89 years, there is growing evidence of DNA methylation and hydroxymethylation changes related to ADNC, especially in the frontal cortex [1–8]. Given the short life expectancy, epigenomic data from people with ADNC over the age of 90 is rare and they are underrepresented in most epigenomic studies. The relation of DNA methylation changes and ADNC at such old age is therefore unknown. Further, AD related methylation changes were so far mainly analyzed using whole-tissue homogenates (‘bulk’) of cerebral cortical regions [1–6] with only a few studies analyzing smaller brain regions thought to be critical to disease progression, like the entorhinal cortex [7, 8]. To develop a more comprehensive understanding of the brain DNA methylome in older individuals, we analyzed bulk samples from eight different brain regions important in the progression of ADNC. Previous studies that analyzed sorted cells instead of bulk samples have shown that cell type proportions of tissue homogenates highly influence methylation changes [5, 9]. Instead of cell sorting, we used cell type deconvolution methods that can extract cell-type-specific (CTS) signals from bulk data [10–12]. Most AD methylome studies focus on the identification of methylation changes related to neurofibrillary tangles or Aβ burden [1–8]. In this study, we focused on methylation changes related to three key measures of ADNC: Aβ plaques, neurofibrillary tangles, and neuritic plaques. We used this approach to identify novel cell-type- and region-specific associations of methylation with ADNC that could not be readily found in bulk data.

## Materials and methods

### Study population

We processed samples from eight brain regions of participants from *The 90*+ *Study*, a longitudinal study of aging and dementia conducted at the University of California, Irvine [13]. Participants were required to be aged 90 years or older to enter the study. Over the course of the study they underwent evaluations including neurological exams and neuropsychological tests every 6 months until the end of life. Approximately 1 in 5 members of this cohort consented to donating their brains for post-mortem research, making a combination of longitudinal clinical and post-mortem neuropathological data available to investigators. We observed low DNA concentrations and poor DNA quality in brain samples from formalin-fixed paraffin-embedded (FFPE) blocks that were prepared before 2017 so we only used consecutive samples prepared between 2017 and 2019. Only cases available to the lab at time of processing were included, cases were not pre-selected based on ADNC scores. Two cases were excluded due to low DNA quality as indicated by high delta CT values in quantitative polymerase chain reaction (qPCR). Four cases were excluded due to non-AD related neuropathology: two cases with a diagnosis of Parkinson's Disease (PD), one case with glioma and one case with extensive bilateral hemorrhage, leading to a total of 53 cases that underwent methylation array processing. We did not exclude individuals based on neuropathological burden of Lewy Bodies, TDP-43 or microvascular lesions. As previously reported, the post mortem interval (PMI) has little effect on DNA methylation [14] so we did not have any exclusion criteria for samples based on PMI.

### Neuropathological assessment and clinical diagnosis

All cases were scored by a neuropathologist following the National Institute on Aging–Alzheimer’s Association (NIA-AA) guidelines for the neuropathologic assessment of Alzheimer’s disease [15, 16]. Neuropathological diagnoses were blinded to all data collected during life, including sex, clinical diagnosis, and neuropsychological test results. We included Aβ plaques (NIA-AA A score), neurofibrillary tangles (NIA-AA B score), and neuritic plaques (NIA-AA C score) in our assessment [15–17]. These scores are not brain region specific. Following the NIA guidelines, an overall AD severity score (ADSS) of not, low, intermediate, or high was determined. For downstream analyses, we grouped individuals with NIA-AA scores of 0 and 1 and individuals with ADSS of not and low. Available clinical information included informant questionnaires [18–20], medical records, longitudinal neuropsychological testing including the Mini-Mental State Examination (MMSE) [21], neurological examinations, and neuroimaging when available. Blinded to pathological evaluation, this combined clinical information was used in a multidisciplinary consensus conference after death (mean time between last clinical assessment and death was 7.1 months) to determine cognitive status following the Diagnostic and Statistical Manual of Mental Disorders 4th edition criteria [22], scoring 0 for normal, 1 for cognitive impairment, no dementia (CIND), and 2 for dementia. Further details on the *90*+ *Study* design and methods can be found in [13, 23].

### Methylation array processing

**Fig.1:**
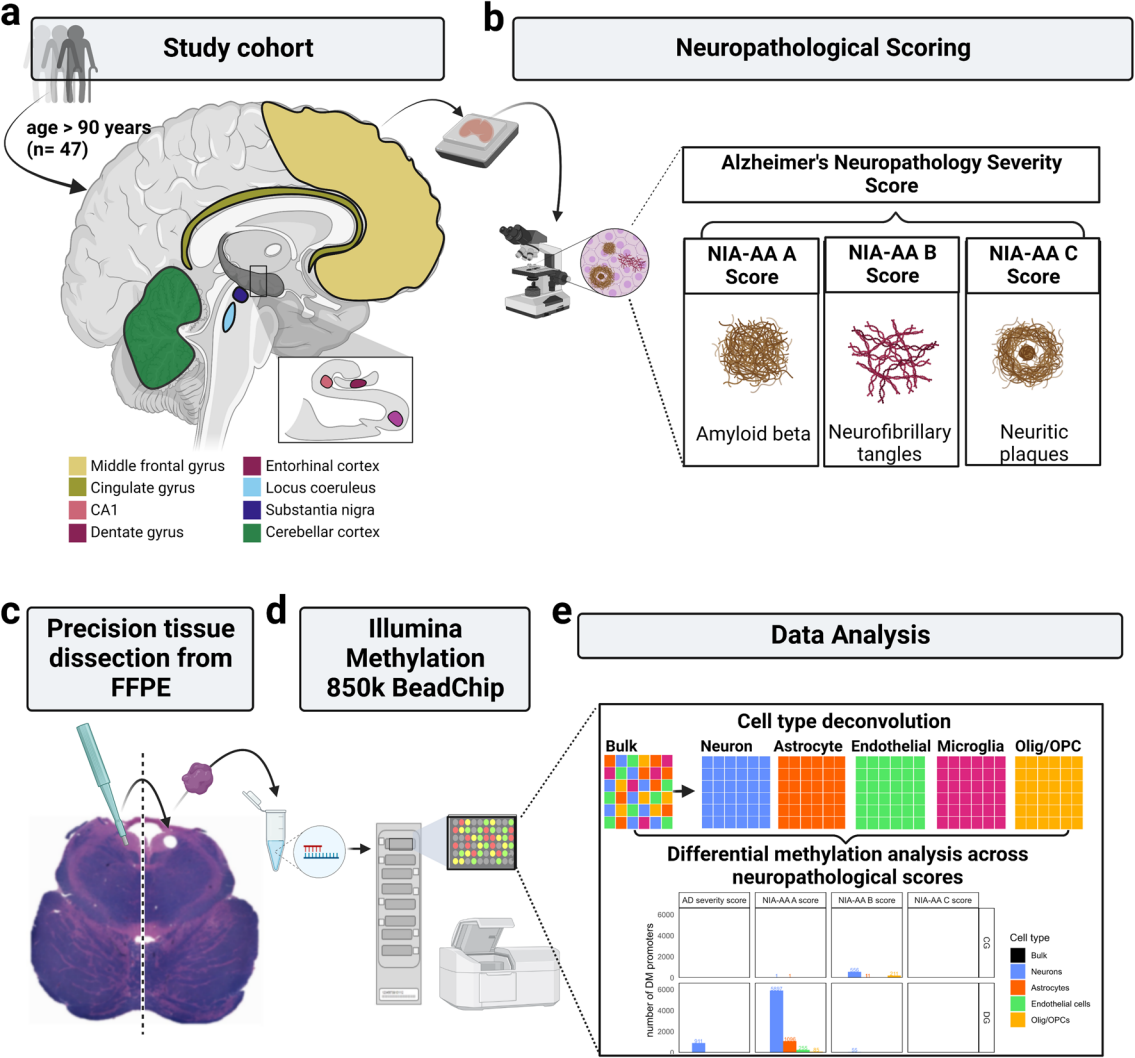
Overview of study concept **a** We selected post mortem Formalin Fixed Paraffin Embedded (FFPE) samples from eight different brain regions from individuals of the *90* + *Study*, aged 90 and older. All consecutive cases available to the lab from 2017 to 2019 were considered. After removing cases based on co-pathologies and data quality, a total of 47 individuals were included in the final analyses for this paper (see Methods). **b** Neuropathological scoring was carried out to define the Alzheimer’s Disease (AD) severity score as well as its three subscores: NIA-AA A (amyloid beta), B (neurofibrillary tangles), and C Score (neuritic plaques). **c** Small biopsy punches were used to assure precision tissue dissection from FFPE. Before and after LFB-H&E staining was carried out to control the accuracy of the dissected region. **d** After DNA extraction and bisulfite conversion, we used the *Illumina 850 k EPIC BeadChip* and followed the manufacturer’s protocol to determine DNA methylation. **e** Data analysis focused on cell type deconvolution from bulk data. *AD* Alzheimer’s disease, *LFB-H&E* Luxol fast blue hematoxylin and eosin, *NIA-AA* National Institute of Aging Alzheimer’s Association, *Olig* Oligodendrocytes, *OPC* Oligodendrocyte Precursor Cells

To focus on brain regions that are most relevant to common neurodegenerative diseases and memory impairment, for each case we dissected samples from the middle frontal gyrus (cortex), cingulate gyrus (cortex), substantia nigra, locus coeruleus, cerebellar cortex and the three hippocampal subregions: CA1, dentate gyrus and entorhinal cortex (Fig. 1a). All samples analyzed in this study were derived from FFPE tissue stored for 0–3 years. Cores were extracted from FFPE blocks with a biopsy punch needle 1–1.5 mm in diameter and 2–3 mm in length, assuring accurate dissection of very fine regions from FFPE. For larger cortical regions, multiple cores were extracted from the same region to ensure pan-cortical coverage. For small regions like the dentate gyrus or locus coeruleus, often only one core could be extracted. To guarantee high regional accuracy, Luxol fast blue hematoxylin and eosin (LFB-H&E) staining was carried out before and after extraction and the accuracy of the dissection was evaluated by a neuropathologist. DNA was extracted from FFPE cores using the *Zymo Research Quick DNA FFPE Miniprep kit* [24] following the manufacturer's instructions with one minor modification. During overnight digestion with proteinase K at 55 °C, intermittent vortexing every 20 min at 2000 rpm for 1 min was used to facilitate more complete digestion of tissue chunks. Double stranded DNA concentration was measured using the *Qubit™* dsDNA BR Assay Kit [25]. Following the manufacturer’s protocol, we used the *Illumina Infinium HD FFPE QC* assay to assess DNA quality prior to bisulfite conversion. Depending on DNA concentration, 250 ng to 1000 ng of high-quality DNA were bisulfite converted using the *Zymo EZ DNA Methylation Kit* (*Zymo Research*, CA, USA). After bisulfite conversion, the entire yield (8 µl) was restored with the Infinium HD FFPE DNA Restore Kit. Samples were further processed as per the manufacturer’s standard protocol for FFPE samples on the *Illumina Infinium Human MethylationEPIC BeadChip* (*Illumina*, CA, USA). We processed up to four arrays simultaneously with 8 samples assayed per array, constituting one processing batch. Samples that underwent the same bisulfite conversion cycle together belonged to the same bisulfite batch. Prior to hybridization, samples were randomized so that samples from one individual were randomly distributed across the arrays. Where possible, samples from one individual were processed in different batches and replicates were also assayed in different batches.

### Data preprocessing

All analysis was carried out in *R* and *python*, using R version 4.2 and python version 3.9.5. Sample-level quality control was performed jointly on samples from all eight brain regions, whereas quality control on a probe level was performed individually within each brain region dataset, as well as the removal of samples based on detection *p*-value > 0.01 in more than 10% of all probes. A flowchart showing the numbers of samples and probes removed for each brain region dataset can be found in Additional File 1: Fig. S1. We assessed sample quality by evaluating bisulfite conversion efficiency as implemented by *minfi* [26], beta value density distribution plots, various *Illumina* control metrics [27] as well as SNP clustering and the fraction of successfully detected probes per sample. Examining each brain region individually, we removed samples that exceeded > 10% of probes with a detection *p*-value > 0.01. Probes were removed if they failed any of these quality control criteria: probes with detection *p*-value > 0.01, probes associated with single nucleotide polymorphisms of minor allele frequency (MAF) ≥ 0.05 [28], cross reactive probes [29, 30], probes with a bead-count < 3 in ≥ 5% of samples as well as probes showing low variability (SD < 0.01) [31]. As our dataset includes male and female participants, we also filtered out probes targeting X and Y chromosomes. Data was normalized using noob implemented by *minfi *[26] and BMIQ [32] implemented by *ChAMP *[33]. We kept the first processed sample and removed all replicates from the datasets after normalization. All samples from six individuals did not pass the described quality control measures, removing the full case from all downstream analyses. The final dataset consisted of 321 samples from 47 individuals. Demographic, clinical, and neuropathological details for all 47 individuals are available in Table 1.

**Table 1:** Characteristics of study participants

Demographics			AD neuropathologies			Copathologies		
Sex			AD severity score			Braak lewy body staging		
	Male	12 (25.53%)		Not	2 (4.26%)		0	31 (65.96%)
	Female	35 (74.47%)		Low	2 (4.26%)		1	4 (8.51%)
Age at death				Intermediate	15 (31.91%)		2	1 (2.13%)
	Mean (SD)	97 (± 3.5)		High	28 (59.57%)		3	3 (6.38%)
Education			NIA-AA A score				4	0 (0.0%)
	Not college	21 (44.68%)		0	2 (4.26%)		5	2 (4.26%)
	Minimum college	26 (55.32%)		1	4 (8.51%)		6	6 (12.77%)
Clinical diagnosis				2	6 (12.77%)	TDP-43		
	Normal	19 (40.43%)		3	35 (74.47%)		0	29 (61.70%)
	CIND	15 (31.91%)	NIA-AA B score				1	4 (8.51%)
	Dementia	13 (27.66%)		2	17 (36.17%)		2	12 (25.53%)
APOE genotype				3	30 (63.83%)		3	2 (4.26%)
	2,3	5 (10.94%)	NIA-AA C score			MVL burden		
	2,4	3 (6.38%)		0	3 (6.38%)		0	38 (80.85%)
	3,3	24 (51.06%)		1	4 (8.51%)		1	5 (10.64%)
	3,4	5 (10.64%)		2	8 (17.02%)		2	1 (2.13%)
	Missing	10 (21.3%)		3	32 (68.09%)		3	3 (6.38%)

Demographics and results of the neuropathological and clinical assessment of all individuals. Categorical variables are displayed as full numbers with percentage (%), continuous variables are displayed as mean with standard deviation (SD). Abbreviations: *AD* Alzheimer’s Disease, *CIND* cognitive impairment, no dementia, *NIA-AA* National Institute of Aging Alzheimer’s Association, *APOE* Apolipoprotein E, *TDP-43* TAR DNA-binding protein 43, *MVL* microvascular lesions

### Cell type deconvolution

To estimate cell type heterogeneity in our bulk data, we used an existing cell-type-specific DNA methylation reference matrix for brain tissue from *EpiSCORE* [12]. *EpiSCORE* is designed to construct reference datasets of tissue-specific DNA methylation derived from single cell RNA-sequencing data. It already contains 13 tissue-specific DNA methylation reference matrices that can be used for cell type deconvolution from bulk data [34]. Bulk data was processed for each brain region separately. First, CpGs were mapped to Entrez IDs using the *constAvBetaTSS* function from the *EpiSCORE* package. Cell type proportions were estimated using the *wRPC* function, using weights in the regression, setting the threshold on the weights to select the most informative genes to 0.4 and the maximum number of iterations in the robust linear regression to 300. The brain reference matrix for *EpiSCORE* can be used to estimate cell type proportions for neurons, astrocytes, endothelial cells, microglia, oligodendrocytes, and oligodendrocyte precursor cells (OPCs). Because we detected relatively small proportions for oligodendrocytes and OPCs and the cell types are highly related, we combined those proportions into one value for oligodendrocytes/OPCs. The resulting estimated cell type proportions for the five cell types served as input for cell type deconvolution.

Cell type deconvolution was performed using the Tensor Composition Analysis (*TCA*) package in *R* [11]. Given the estimated cell type proportions, *TCA* uses a tensor generalization of matrix factorization to estimate CTS methylation profiles for each of the five cell types we considered. The resulting deconvolved data consists of a CTS methylation value for each CpG and sample. We deconvolved the five cell types within each of the eight brain regions separately producing forty datasets for downstream analyses. Within each CTS methylation profile, we collapsed probe-level methylation to three genomic region types (promoters, genes, and CpG islands) using the *RnBeads* annotations [31], by calculating the average methylation value across all CpGs within the respective genomic region. A promoter was defined as the region spanning 1500 bases upstream and 500 bases downstream of the transcription start site of the corresponding gene [31]. Gene bodies were defined as the region from the transcription start site to the end of 3’ UTR, and CpG islands in *RnBeads* are downloaded from the *UCSC Genome Browser* [31]. Together with CpG-level methylation, these three regional summaries resulted in four datasets for each combination of brain region and cell type.

### Batch correction

We found that batch was significantly confounded with neuropathology and clinical measures using the Kruskal–Wallis test for continuous outcomes and the G-Test for categorical outcomes (Additional File 2). Because the batches in our study were small when considering an individual brain region, supervised removal of the batch effects directly with linear regression performed poorly because of high uncertainty in estimating the effects of individual batches. Instead, we used singular value decomposition (SVD) to identify surrogate variables that capture the variance associated with batch, similar to approaches used in previous studies [35]. In each brain region, we used the inverse logit transformation implemented in *RnBeads* to convert methylation beta values to M-values [31], which gives them a Gaussian distribution more suitable for downstream analyses such as SVD and linear regression [36]. We used SVD to obtain eigenvectors and eigenvalues that capture global signals shared across all CpGs, which can include batch effects, cell type heterogeneity and disease pathology. The intrinsic dimensionality of each dataset was estimated using the *EstDimRMT* function from the *iSVA* R package, which uses the Marcenko-Pastur distribution [37] to determine how many eigenvectors have eigenvalues that exceed those expected from Gaussian white noise. Datasets with an estimated dimensionality less than six were excluded from downstream analyses to avoid spurious results (34 datasets excluded). These excluded datasets were mainly represented by regions with low estimated cell counts for microglia and in the cerebellar cortex, astrocytes and oligodendrocytes/OPCs (Additional File 3). The association of each eigenvector with pathology scores, clinical measures and batch variables was tested using the non-parametric Kruskal–Wallis or Spearman rank correlation tests for categorical or numeric variables, respectively. We adjusted each dataset for all of its corresponding eigenvectors using residualization with the *removeBatchEffect* function from *limma* [38], except for eigenvectors which were significantly associated with pathology scores or clinical measures and not with batch. This approach removes technical effects from batch, while preserving disease-associated signals if they are not confounded with batch.

### Data visualization

We used the Uniform Manifold Approximation and Projection (UMAP) algorithm to visualize the two-dimensional embedding of our bulk and CTS methylation profiles from each brain region. As input to UMAP, we computed principal components on the M-values of the combined set of bulk and CTS methylation profiles, using the Marcenko-Pastur distribution [37] to choose the number of PCs as previously described. We used the *uwot* package to estimate the UMAP embeddings for the principal components and visualized the embedding using *ggplot2 *[39].

### Differential methylation analysis

To explore brain region and cell-type-specific methylation differences associated with Alzheimer's disease, we examined both neuropathological scores as well as clinical measures (see Methods section “Neuropathological assessment and clinical diagnosis”). Neuropathological scores were defined following the NIA-AA guidelines and were case specific, but not brain region specific. We used *limma* [38] to fit linear models for differential methylation analysis, treating the neuropathological scores as continuous variables and the clinical diagnosis as a categorical variable. Sex and age at death were added as covariates to the linear models. To account for the removal of variables in the batch correction step, we subtracted the number of eigenvectors removed from the sample during batch correction from the residual degrees of freedom used to compute the t-statistics. We considered sites or summarized regions with an empirical Bayes False discovery rate (FDR) adjusted *p*-value < 0.05 and an absolute log fold change (logFC) ≥ 0.0001 to be differentially methylated. We used *biomaRt* [40] to annotate gene biotypes of promoter regions and gene bodies to identify protein-coding genes.

### Immunohistochemistry (IHC) staining

Sections were derived from FFPE blocks, baked for 1 h at 70 °C, then deparaffinized and rehydrated with 1-min washes in xylene (3 ×), 100% ethanol (2 ×), 95% ethanol (2 ×), 80% ethanol (1 ×), 70% ethanol (1 ×) and H2O (3 ×). Antigen retrieval was carried out in citrate buffer pH 6.0 (*Dako*, cat# S1699) at 95 °C for 25 min and allowed to cool to room temperature for 40 min. Slides were then washed twice for 5 min each in PBS-T IHC Wash Buffer (*Cell Marque*, cat# 934B-09) with 0.1% bovine serum albumin. Endogenous peroxidase activity was quenched for 30 min with 3% H2O2. Sections were washed with a wash buffer for 5 min and then blocked for 1-h at room temperature in TBS-T with 3% normal horse serum, 0.1% cold fish gelatin, 0.1% triton x-100, and 0.05% sodium azide. Primary antibody (*PSENEN*, *Sigma-Aldrich*, cat# HPA047435) for Presenilin Enhancer 2 (*PEN-2*) was used at 0.5ug/mL and diluted with 3% normal horse serum and incubated overnight at 4 °C. Following overnight incubation, slides were washed twice for 5 min, and sections were incubated in ImmPRESS HRP Horse Anti-Rabbit IgG Peroxidase (*Vector Laboratories*, cat# MP-7401) for 30 min at room temperature. Slides were then washed twice for 5 min each and subsequently developed using the ImmPACT DAB kit (*Vector Laboratories*, cat# SK-4105). Tissue was counterstained with hematoxylin and blued with Scott’s Tap Water Substitute prior to dehydration and mounting with Mounting Media (*Thermo-Scientific* cat# 22-110-610).

## Results

We analyzed DNA methylation in eight brain regions from a unique cohort of participants aged 90 years and older in order to identify DNA methylation differences related to the endophenotypes of the three hallmark neuropathologic lesions of AD (Fig. 1a, b). Using the *Illumina 850k* platform, we assayed methylation of 853,307 CpGs in eight regions of the human brain: middle frontal gyrus (MFG), cingulate gyrus (CG), entorhinal cortex (EC), hippocampus dentate gyrus (DG), hippocampus CA1 (CA1), substantia nigra (SN), locus coeruleus (LC) and cerebellar cortex (CBM). Computational cell type deconvolution was used to investigate differential methylation in CTS data (Fig. 1d, e).

### Characteristics of study participants

After removing samples from individuals with failed methylation array processing (see Methods), our final cohort consisted of 321 samples from 47 individuals (Fig. 1a). Eight brain regions were processed from each individual. Characteristics of study participants are shown in Table 1. The mean age was 97.4 ± 3.5 years, with little difference in age observed between sexes. The majority of individuals were females (n = 35), reflecting the overall demographics of people aged over 90 [41]. Amongst all individuals, 17.32% are heterozygous carriers of the *APOE* ɛ4 allele; data from 10 individuals were missing due to problems in sequencing. Concerning clinical diagnosis, 40.43% of our participants were diagnosed as cognitively normal, 31.9% had cognitive impairment, no dementia (CIND) and 27.66% were diagnosed with dementia. Only 4 individuals (8.51%) had an overall low neuropathological burden of AD as measured by the AD severity score (see Methods), 15 were classified as intermediate (31.91%) and 28 individuals had a high burden of AD neuropathology (59.57%). Detailed demographics and neuropathological data for each case can be found in Additional File 4.

### Computationally deconvoluted cell type proportions unveil regional differences

We estimated cell type proportions of our samples using a cell type decomposition method that utilizes an existing reference panel from *EpiScore* [12] (see Methods). As expected, neurons were the most abundant cell type in most brain regions (Additional File 1: Fig. S2) and predicted proportions were consistent with observations from previously published epigenome wide association studies [2, 7, 42–45]. Cell composition varied between brain regions, with the highest proportion of neuronal signal in the cerebellar cortex, followed by regions in the forebrain, midbrain, and finally pons. Similar to previous findings, the proportion of neurons also varied within the same brain region between individuals (Additional File 1: Fig. S2) [46, 47]. Microglia were the least abundant glial cell type, only being detected consistently in SN and LC. Microglia account for 0.5%-16.6% of cells across brain regions, with the pons and basal ganglia showing higher amounts of microglia than cerebral cortical regions [48], but our average proportions of 3.2% ± 4.4 in the SN and 6.8% ± 5.1 in LC were modestly lower than previously reported proportions greater than 10% [48]. Our predicted proportions of oligodendrocytes/OPCs varied from 0% (in the cerebellum) to up to ~ 40% and were consistent with proportions found in the adult mouse brain (0–40%) [49]. The low estimated proportion of glial cells in the cerebellum also matched previous findings [50]. Similar to existing literature, we did not find any significant correlations of cell composition with neuropathological traits or age at death [42–45].

### Cell-type-specific methylation profiles differ across brain regions

**Fig. 2:**
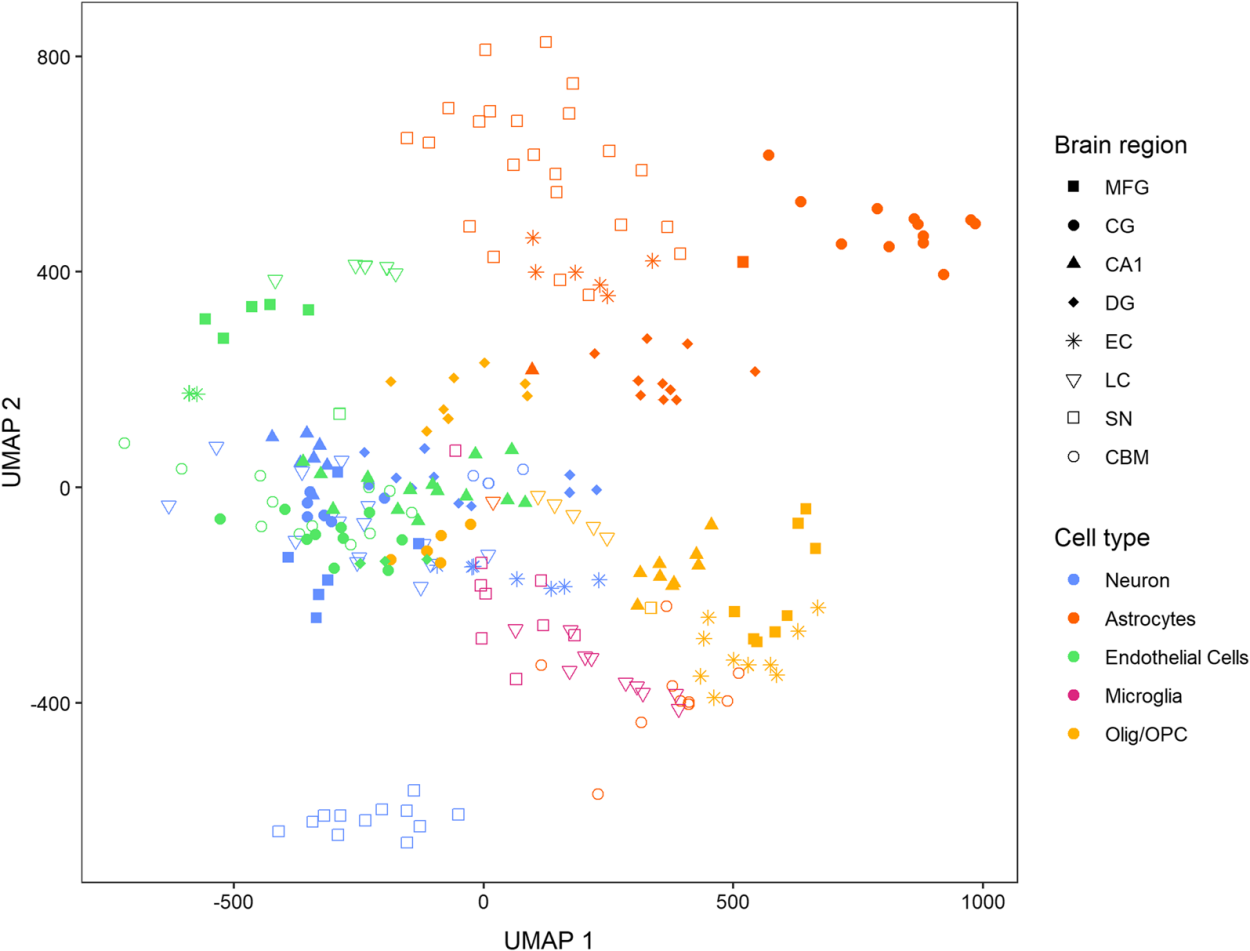
UMAP plot displaying clustering of brain region and cell-type-specific methylation data. We used the Uniform Manifold Approximation and Projection (UMAP) technique for dimension reduction to visualize similarities across cell type and brain-region-specific methylation data. Dimensionality reduction is performed with all brain regions and cell types combined. Each dot represents one individual sample. Colors reflect the cell types. *Olig* Oligodendrocytes, *OPC* Oligodendrocyte Precursor Cells, *MFG* Middle Frontal Gyrus, *CG* Cingulate Gyrus, *CA1* Hippocampus CA1, *DG* Dentate Gyrus, *EC* Entorhinal cortex, *SN* Substantia nigra, *LC* Locus coeruleus, *CBM* Cerebellar cortex

We recovered CTS methylation signals using cell type deconvolution with *TCA* [11], which relies on the cell type proportions estimated with *EpiScore* [12] (see Methods). Although these CTS profiles were imputed from bulk data and not obtained through sorting, for simplicity, we will refer to them as data from their respective cell types. In addition to analyzing the CpG level data, we also aggregated our deconvolved data by averaging methylation across individual CpGs within promoters, gene bodies and CpG islands. We used principal component analysis (PCA) and random matrix theory to identify CTS methylation profiles with sufficiently non-random signals. We computed the full set of eigenvectors and eigenvalues for each cell type and aggregation type, and excluded CTS profiles where the number of eigenvalues which exceeded the theoretical limit expected under the Marcenko-Pastur distribution [37] for a matrix of random noise was less than or equal to 5 (Additional File 3). Most microglial profiles were excluded, except in the LC and the CpG and gene level data in the SN. We further excluded all datasets of oligodendrocytes/OPCs and astrocytes in the CBM, and the CpG islands dataset of astrocytes in the EC. Except for astrocytes in the EC, these profiles corresponded to cell types with very low average estimated proportions in their respective regions, ranging between 0 and 1.2%. We used the UMAP algorithm [51] to visualize the latent space in two dimensions across CpGs for all cell types and brain regions (Additional File 1: Fig. S3) and found that the bulk profiles from different brain regions embedded closely together relative to the CTS profiles, indicating high homogeneity across regions. With the exception of nigral neurons, neuronal profiles were embedded in proximity to bulk data, reflecting the predominance of the neuronal signal in bulk data. The clear separation of CTS methylation profiles from bulk profiles shows that cell type deconvolution is an effective strategy for disentangling signals from distinct cell types in tissue homogenates (Additional File 1: Fig S3). Figure 2 displays a UMAP plot of CTS profiles across brain regions and cell types and visualizes that cell type and not brain region is the main driver of variance in our data. Neurons and astrocytes of the SN were embedded further from similar cell types in other brain regions. Among the non-neuronal CTS profiles (astrocytes, oligodendrocytes/OPCs, microglia and endothelial cells), we found that profiles from the same cell type were usually embedded more closely to each other than to other samples from the same region (Fig. 2). This observation is broadly consistent with findings reported in single cell ATAC-Seq data, where non-neuronal cell types showed greater homogeneity across regions than neurons [52].

### Cell type deconvolution uncovers ADNC related methylation differences not present in bulk data

**Fig. 3:**
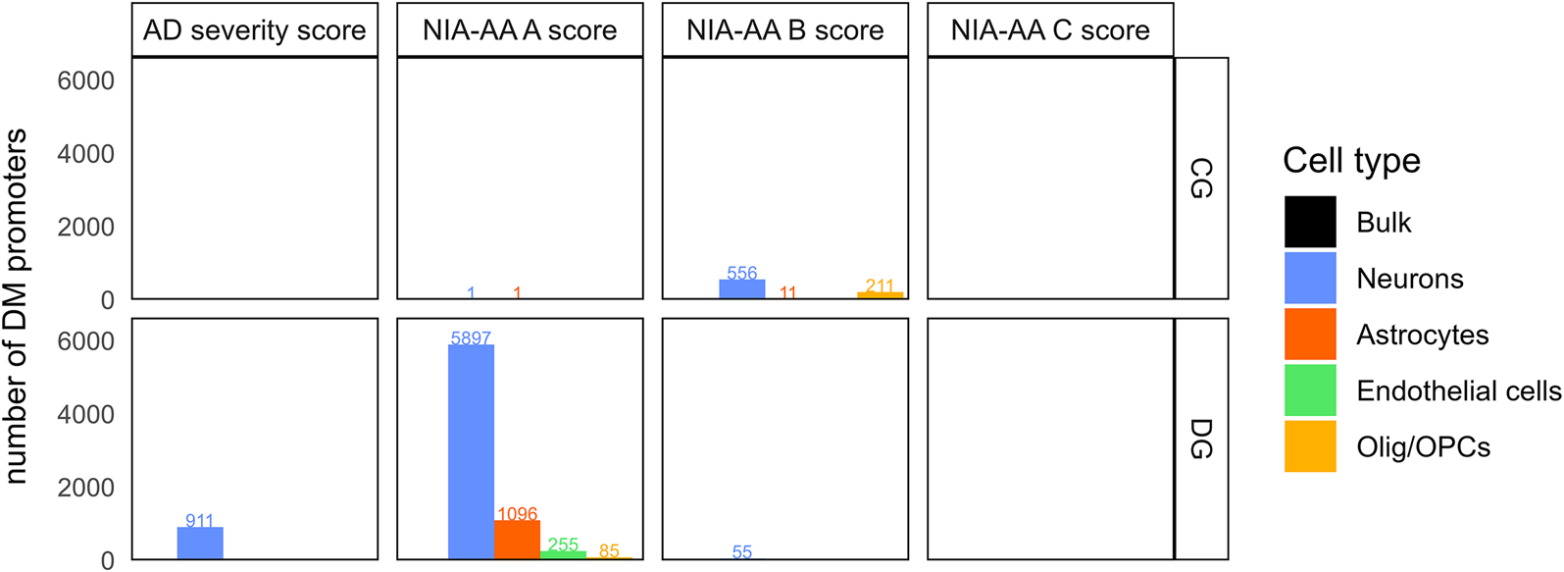
Overview of the number of differentially methylated promoter-associated protein-coding regions for dentate gyrus and cingulate gyrus by cell type. Barplots display the number of significant (FDR *p*-value < 0.05) differentially methylated promoter regions of protein-coding genes. Each barplot shows the results for one brain region and neuropathological score combination. Color coding of the bars reflects the different cell types. The dentate gyrus (DG) and cingulate gyrus (CG) were the two regions with the highest amount of differentially methylated promoters (DM promoters, DMPTs) across neuropathological scores. We did not discover any DM promoters across different NIA-AA C scores, and there were no differentially methylated sites found in bulk data. *FDR* False discovery rate*, Olig* Oligodendrocytes, *OPCs* Oligodendrocyte Precursor Cells, *CG* Cingulate Gyrus, *DG* Dentate Gyrus, *NIA-AA* National institute of Aging Alzheimer's Association, *AD* Alzheimer’s Disease

We used differential methylation to associate bulk and CTS methylation profiles with ADNC endophenotypes in all brain regions and clinical measures (see Methods). We chose to highlight results for associations with averaged promoter methylation in protein-coding genes because these associations are more readily interpretable. Generally, promoter methylation is negatively correlated with gene expression, and limiting the analysis to protein-coding genes avoids concerns about poorly-annotated gene models for non-coding genes. We found no association between ADSS and bulk methylation in any brain region (Additional File 1: Fig. S4). In contrast, numerous associations were found between ADSS and CTS profiles, especially in the dentate gyrus (DG) where we identified 911 differentially methylated promoters (DMPTs) in neurons, all of which were unique to the DG (Fig. 3).

We wanted to perform a more granular analysis of the association of ADNC endophenotypes with methylation by examining differential methylation for Aβ plaques (NIA-AA A score), neurofibrillary tangles (NIA-AA B score) and neuritic plaques (NIA-AA C score). No associations were seen between the individual scores and bulk methylation profiles in any region except for 2 DMPTs associated with NIA-AA C score in the cerebellum. In the CTS profiles, the largest numbers of DMPTs were found in neurons of the CG in association with neurofibrillary tangles and in neurons of the DG in association with amyloid burden (Fig. 3). The DG was the only brain region where NIA-AA A score was associated with a substantial number of DMPTs in neurons (n = 5897 DMPTs), astrocytes (n = 1096 DMPTs), endothelial cells (n = 255 DMPTs), or oligodendrocytes/OPCs (n = 85 DMPTs). Another 4 DMPTs associated with NIA-AA A score were found in neurons of the MFG.

**Fig.4:**
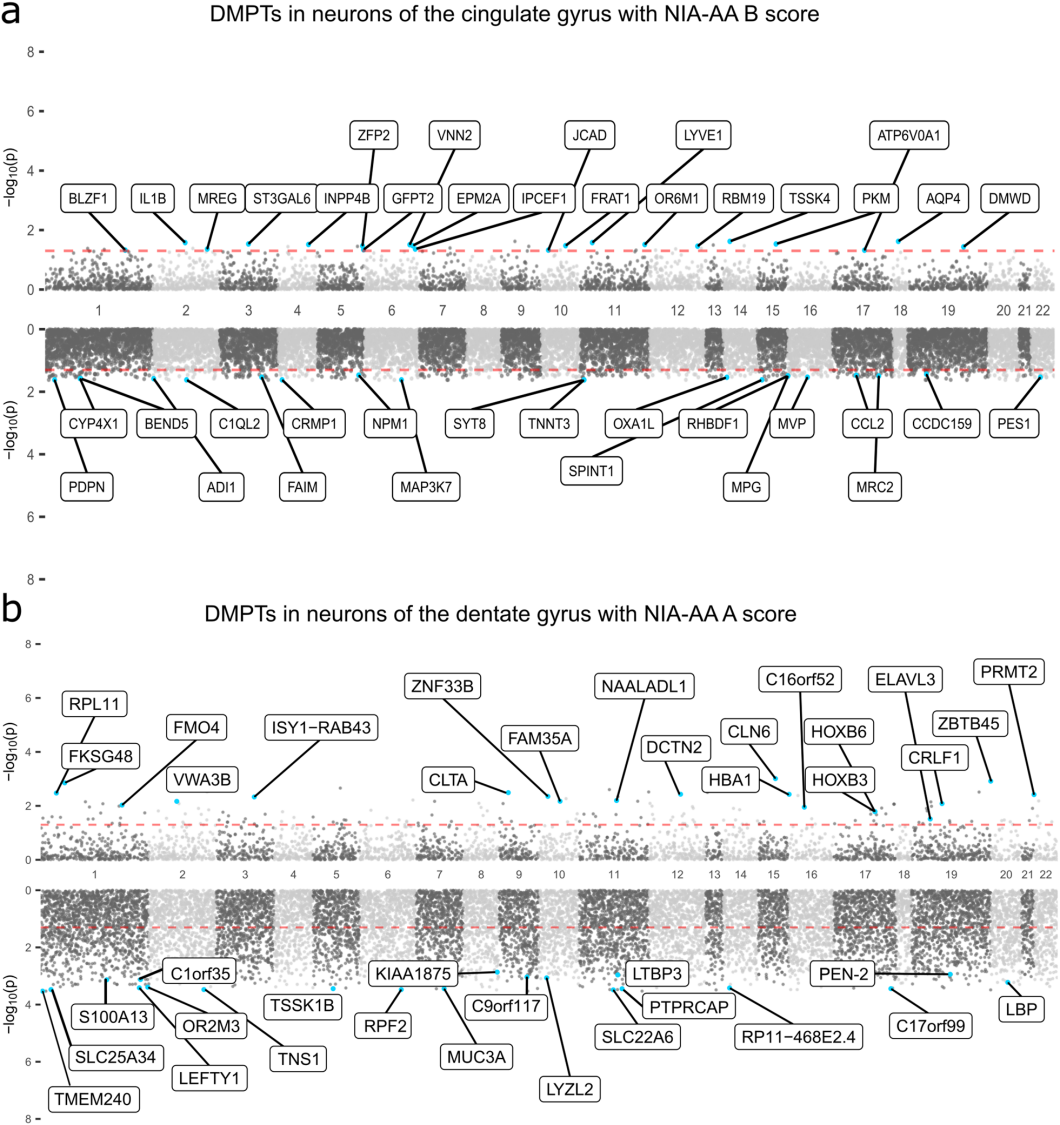
Manhattan mirror plot of differentially methylated promoter regions (DMPTs) in the dentate gyrus. Manhattan plots visualizing **a** the results from differential methylation analysis in promoter associated regions in neuronal signals of the dentate gyrus across NIA-AA A scores (Aβ plaque burden) and **b** the results from differential methylation analysis in promoter associated regions in neuronal signals of the dentate gyrus comparing individuals with different NIA-AA B scores (neurofibrillary tangle burden). Each dot represents the averaged methylation across all CpGs within a specific promoter region. The top part of each plot contains all promoters that are hypermethylated with **a** higher Aβ plaque burden or **b** higher burden of neurofibrillary tangles, and the bottom plot respectively shows all promoters that are hypomethylated. The x-axis displays chromosomes from 1 to 22 from the left to the right. The y-axis is displaying the-log10 FDR p-value as a significance measure for the methylation difference across neuropathological scores. The red dotted line marks the significance threshold of *p* < 0.05. The blue dots highlight the top 20 significant protein-coding promoters as ranked by log fold change of the methylation beta value. Labels display the name of the associated protein-coding gene for each of the top 20 differentially methylated promoters. *DMPT* differentially methylated promoter region, *NIA-AA* National institute of Aging Alzheimer's Association

The NIA-AA B score, a measure of neurofibrillary tangle (NFT) burden, is most commonly used as a measure of AD neuropathology in epigenome-wide association studies (EWAS) of AD. In our study, the CG showed the highest number of DMPTs in association with neurofibrillary tangles, found mainly in neurons (n = 556) but also in astrocytes (n = 11) and oligodendrocytes/OPCs (n = 211) of the CG (Fig. 3). Only a few DMPTs were found in neurons of the DG (n = 55) and no other brain regions showed significant DMPTs in association with neurofibrillary tangles. The DMPTs of neurons in the GG are visualized in Fig. 4a, with the top 20 hyper-and hypomethylated genes labeled accordingly. Most differentially methylated promoter regions were hypomethylated with increasing NIA-AA B score.

For the NIA-AA C score, only a small number of DMPTs were found in microglia of the LC (n = 3, Additional File 1: Fig. S4), and no significant DMPTs were identified in the other brain regions (CBM, EC, SN, CA1).

We examined several copathologies and did not find any significant DMPTs associated with TDP-43 burden or Braak staging for Lewy bodies in any of the brain regions in the CTS profiles (Additional File 1: Fig. S5). We found very few significant (FDR p < 0.05) DMPTs associated with microvascular lesions in neurons of the locus coeruleus (n = 5, *F2*, *CISD2, WFDC6, LYPLAL1, GPR33*) and substantia nigra (n = 1, *KLHL14* hypomethylation, logFC = 0.70) and in astrocytes of the locus coeruleus (n = 1, *PPP1RC* hypermethylation, logFC = 0.58) and entorhinal cortex (n = 1, *SCML4* hypomethylation, logFC = 0.60). One DMPT was associated with microvascular lesions in oligodendrocytes/OPCs of the substantia nigra (*MYL6B* hypermethylation, logFC = 0.84).

### Hypomethylation at promoter regions of known AD risk loci with increasing burden of AD neuropathology is mainly found in neurons of the dentate gyrus

We identified several significantly hypomethylated promoters among the top 33 AD risk loci previously identified in AD GWAS studies [53]. In neurons of the CG, the promoter region of the *UNC5CL* gene was hypomethylated with increasing burden of neurofibrillary tangles (logFC = 0.71, FDR *p* < 0.05). In astrocytes of the CG, promoter regions of the *SPI1* and *CR1* genes were hypomethylated with increasing burden of amyloid plaques (*SPI1* logFC = 0.56, *CR1* logFC = 0.55, FDR *p* < 0.05). In neurons of the DG, 12 out of the top 33 AD GWAS risk loci were hypomethylated with increasing amyloid plaque burden (*SPI1* logFC = 0.53, *WNT3* logFC = 0.64, *CLNK* logFC = 0.37, *CLU* logFC = 0.46, *UNC5CL* logFC = 0.31, *BIN1* logFC = 0.36, *SORL1* logFC = 0.37, *IL34* logFC = 0.52*, ACE* logFC = 0.45*, INPP5D* logFC = 0.59, *PLCG2* logFC = 0.49, *CD2AP* logFC = 0.27; FDR *p* < 0.05). *CLU* and *ACE* promoter hypomethylation was also seen in association with the overall AD Severity Score in neurons of the DG (*CLU* logFC = 0.42, *ACE* logFC = 0.38, FDR *p* < 0.05).

### Promoter hypomethylation of *PEN-2* with increasing burden of amyloid plaques is unique to neurons of the dentate gyrus

**Fig. 5:**
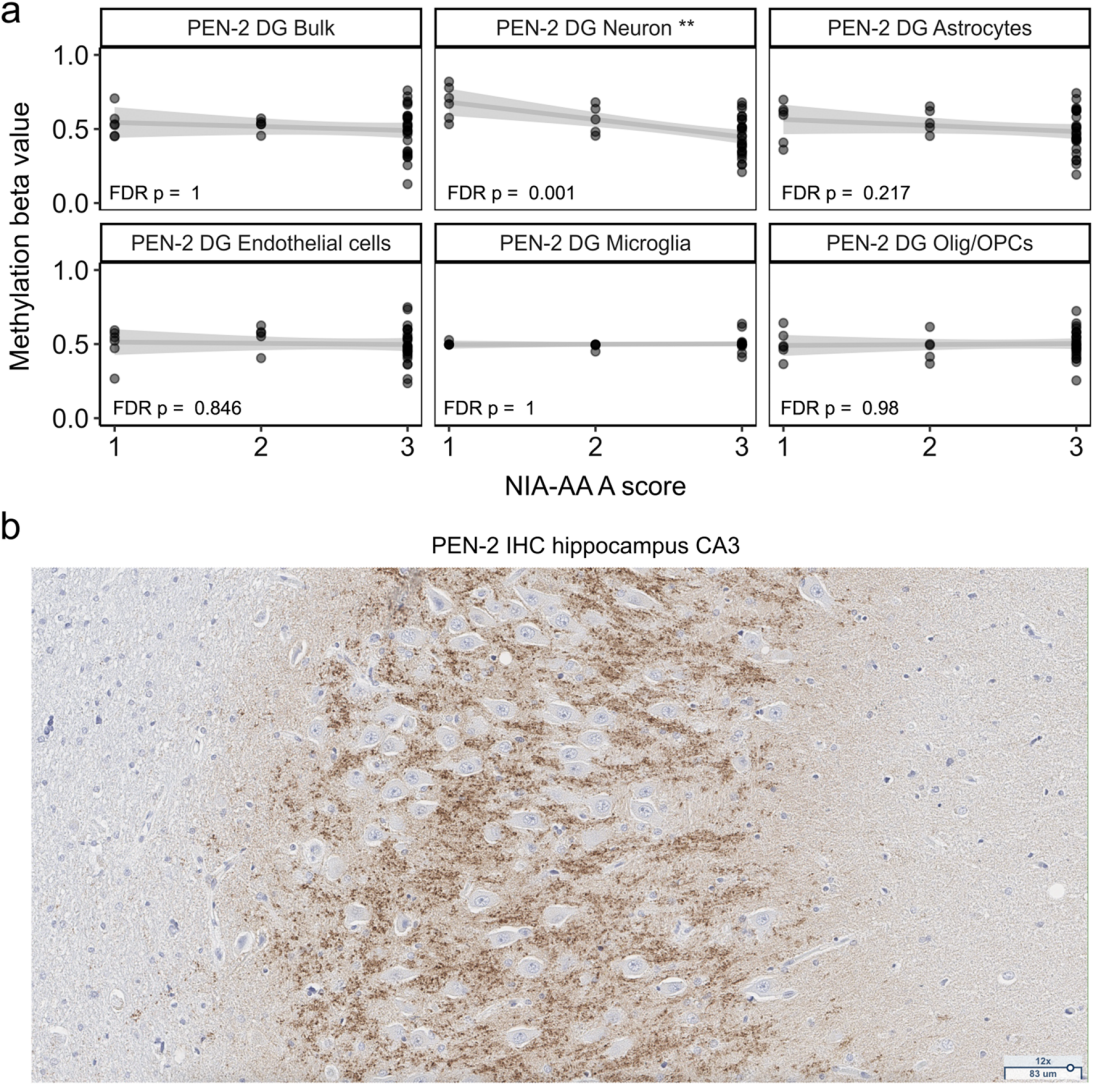
Cell-type-specific promoter methylation of the *PEN-2* gene in dentate gyrus (DG) across individuals with different NIA-AA A scores. **a** Scatterplots with smoothers showing the relationship between neuronal methylation of the promoter region of the *PEN-2* gene (y-axis) across the five different cell types and bulk data from individuals with different Aβ plaque burden (NIA-AA A scores, x-axis). Methylation beta values are displayed on the y-axis and the categories of the NIA-AA A score on the x-axis. Each individual plot shows data from the dentate gyrus (DG) for different cell types. Each dot represents one individual sample. The standard linear regression was plotted as smoothers on top of the data: Smoothers curves are showing the relationship (solid line) between the NIA-AA A score and the methylation beta-value. Shaded areas indicate the 95% confidence interval of the smooth curve. We saw significant hypomethylation (**FDR *p* = 0.001, logFC = 0.80) in the promoter region of the *PEN-2* gene with increasing Aβ plaque burden in the dentate gyrus. **b** Immunohistochemistry (IHC) of *PEN-2* in the hippocampal region Cornu Amonis 3 (CA3) of an individual with high Alzheimer’s disease neuropathological changes (AD severity score = 3). IHC Scoring 3+ . The respective participant was *Participant 44*; for extended phenotype data of this individual see Additional File 4*.* *Olig/OPCs* Oligodendrocytes/Oligodendrocyte Precursor Cells. *DG* Dentate gyrus, *Aβ* Amyloid beta

We identified the largest number of DMPTs in the DG, predominantly in neurons, but also in astrocytes, endothelial cells and oligodendrocytes/OPCs (Fig. 3). All 911 DMPTs found in neurons were specific to the DG. The DG has been underrepresented in methylation studies of the human brain, prompting us to explore further the DMPTs associated with NIA-AA A score in this region. The results are visualized in Fig. 4b, with the top 20 hyper- and hypomethylated promoter regions labeled accordingly. Similar to DMPTs associated with NIA-AA B score in the CG (Fig. 4a), DMPTs associated with increased Aβ plaque load in promoter regions of neurons of the DG were mostly hypomethylated (Fig. 4b). This also holds true for global methylation at overall non-averaged CpGs. We saw a negative correlation of global methylation levels with NIA-AA A score in neurons (spearman rho =  − 0.58, FDR *p* = 0.005), astrocytes (rho =  − 0.49, FDR *p* = 0.019) and microglia (rho =  − 0.55, FDR *p* = 0.007) in DG. These results are consistent with previous findings where overall neuronal DNA-methylation in the hippocampus was negatively correlated with AD burden [54]. The top 20 DMPTs (based on log fold change (logFC)) found in the DG included several genomic regions that are known to be altered in AD [55–58] like presenilin enhancer 2 (*PEN-2*, logFC =  − 0.80, FDR *p* = 0.001), solute carrier family 22 member 6 (*SLC22A6*, logFC = -0.94, FDR *p* < 0.001), lipopolysaccharide binding protein (*LBP*, logFC =  − 0.81, FDR *p* < 0.001), and S100 calcium binding protein A13 (*S100A13*, logFC =  − 0.81, FDR *p* < 0.001). A complete summary of DMPT statistics is available in Additional File 5. Across these four genes, promoter hypomethylation in neurons with increasing burden of Aβ plaques was unique to the dentate gyrus (Additional File 1: Fig. S6). Focusing on *PEN-2*, hypomethylation was also significantly associated with ADSS (FDR *p* = 0.016, Additional File 1: Fig. S7), reflecting the influence of the amyloid burden in this aggregated score. In the DG, the association of NIA-AA A score with *PEN-2* hypomethylation was only found in neurons, and, crucially, could not be identified in the bulk methylation profile (Fig. 5a). As promoter methylation is often expected to reduce gene expression [59–61], we could expect the hypomethylation we observed to lead to higher expression of *PEN-2* and perhaps contribute to proteolytic processing of amyloid precursor protein along the gamma(γ)-secretase pathway [62, 63]. Indeed, downregulation of the *PEN-2* gene directly impairs γ-secretase activity, and overexpression has been found to increase activity of the γ-secretase [64, 65]. Other subunits of the γ-secretase complex are the presenilins (*PSEN1* and *PSEN2*), aph-1 homolog A (*APH-1A)* and nicastrin (*NCSTN*). Numerous mutations in *PSEN1* and *PSEN2* have been identified in cases of familial early onset AD [66], but less is known about the regulation of the γ-secretase complex in late onset AD. Among the four subunits of the γ-secretase complex, in addition to *PEN-2*, hypomethylation of *NCSTN* was also associated with higher Aβ plaque burden in the DG neuron profile (Additional File 1: Fig. S8).

### Immunohistochemistry staining for *PEN-2* shows regional expression in the dentate gyrus and substantia nigra

In our cohort, the hypomethylation of *PEN-2* with an increasing burden of Aβ plaques was unique to neurons of the dentate gyrus. As there is little known about the regional expression of *PEN-2* in the human AD brain, we conducted immunohistochemistry (IHC) staining on one case with high ADNC (AD severity score of 3) across all eight brain regions that were prior sampled for methylation analysis. The positivity for *PEN-2* by IHC was strongest in the hippocampus (Fig. 5b) and substantia nigra with robust positivity and very specific staining, followed by the periaqueductal gray with intermediate positivity, and cingulate and middle frontal gyrus both showed the weakest positivity with diffuse staining in the gray matter. Cerebellum and pons were both negative (Figures not shown). IHC is a localizing and not a robustly quantitative technique because of pre-analytic and analytical variability [67]. Furthermore, the correlation between gene methylation and protein abundance is poor, reflecting the multiple layers of regulation between these two events. With these cautions in mind, we performed *PEN-2* IHC on the hippocampus of two cases with high and two cases with low ADNC and observed variation across cases that was independent of ADNC status (Additional File 1: Fig. S9). Nevertheless, our small IHC experiment showed that in a brain with ADNC, *PEN-2* was highly expressed in the hippocampus and substantia nigra. In contrast to findings in mouse models of AD, we did not see expression in cerebellum and pons [68].

### Differential promoter methylation of clathrin-mediated endocytosis genes associates with increasing burden of Aβ plaques in neurons of the dentate gyrus

In addition to findings in genes of the γ-secretase complex, amongst the top 20 DMPTs in neurons of the dentate gyrus, the promoter region of clathrin light chain A (*CLTA*) was hypermethylated with increasing burden of Aβ plaques (Fig. 4b). *CLTA* encodes one of two clathrin light chain proteins, which form part of the regulatory function of the clathrin protein, an important protein for endocytotic processes in synaptic trafficking [69]. Clathrin-mediated endocytosis (CME) of amyloid precursor protein is of great relevance to AD pathology because it impacts the production of Aβ [70, 71]. Although not amongst the top 20 DMPTs, we found two more genes that are involved in CME to be significantly (FDR *p* < 0.05) hypomethylated with increasing amyloid burden: *BIN1* and *CD2AP*. These genes were previously found to be associated with AD in both epigenome and genome wide association studies [2, 72–75]. In our study, the differential methylation of the promoter regions of *CLTA, CD2AP and BIN1* with increasing amyloid burden was unique to neurons of the dentate gyrus and could not be found in any other brain region or cell type (Additional File 1: Figs. S10, S11). Notably, we did not see any association with AD neuropathological scores other than amyloid plaque burden.

### Differential methylation in the dentate gyrus is related to neuropathology but not cognitive performance

In addition to neuropathologic changes, we assessed whether methylation differences were also associated with cognitive performance in old age. We tested differential methylation at promoters of protein-coding genes across individuals with one of three cognitive statuses: normal, cognitive impaired not dementia (CIND), or dementia. In contrast to the results from neuropathologic features, we did not detect any DMPTs in the dentate gyrus. The ERC was the only region showing cell-type-specific methylation differences across clinical groups, with a small number of DMPTs found in neurons (n = 6) distinguishing cognitively normal from demented individuals and in astrocytes (n = 6, Additional File 1: Fig. S12) distinguishing individuals with CIND and normal cognitive status, none of which overlapped. Further, treating the clinical status as a continuous rather than a categorical variable yielded no significant associations that might indicate a continuous methylation change related to cognitive decline.

## Discussion

With this dataset, we provide the most detailed methylation microarray-based study of the oldest old human brain to date, providing a valuable reference for future studies on brain aging and neurodegeneration. Our precise dissection protocol enabled the study of smaller brain regions that have been implicated in neurodegenerative diseases but are rarely studied at such a granular scale. Notably, this is the first methylation study specifically examining small brain regions like the locus coeruleus, substantia nigra, and the hippocampal subregions CA1 and dentate gyrus (DG). Prior studies of methylation in AD have mainly focused on cortical regions [1–6] or analyzed the entire hippocampus [76], treating the CA1, entorhinal cortex and dentate gyrus as a single entity, while we analyzed them separately. We showed that “digital sorting” with cell type deconvolution can uncover methylation signals that are otherwise obscured in bulk data, suggesting that cell type heterogeneity should always be considered when interpreting the results of methylation studies in bulk tissue. Within our CTS profiles, neurons of the cingulate gyrus and neurons of the dentate gyrus had the largest number of associations between methylation and increased burden of ADNC. In neurons of the cingulate gyrus, promoter methylation was mainly decreased with increasing burden of neurofibrillary tangles. Amongst others, *UNC5CL* as a known risk gene for AD was hypomethylated with increased burden of neurofibrillary tangles. In neurons of the dentate gyrus, a substantial amount of known AD risk loci were hypomethylated at promoter regions with increasing Aβ burden. Since we did not make as many discoveries in other brain regions or cell types, we focused our further analyses on neurons of the dentate gyrus. In particular, two components of the γ-secretase complex, *PEN-2* and *NCSTN*, were hypomethylated with increased Aβ burden (Additional File 1: Fig. S8). *PEN-2* is known to be the rate limiting protein for the formation of the γ-secretase complex, initiating the sequential endoproteolytic cleavage of amyloid precursor protein into amyloid β [63]. A *PEN-2* missense mutation is linked to familial AD in humans [77], knockdown of *PEN-2* in zebrafish leads to neuronal loss and apoptosis [78], and loss of *PEN-2* causes astrogliosis, enhanced inflammatory responses, age-dependent cortical atrophy, and neuronal loss in mice [79]. Our small IHC experiment showed a regional expression of *PEN-2* in the hippocampus and substantia nigra in a participant with high ADNC. As multiple mechanisms other than methylation can impact gene expression, we cannot predict the direct impact of *PEN-2* and *NCSTN* promoter hypomethylation on the activity of their respective proteins or the γ-secretase complex and further investigations will be needed to validate and better understand this novel discovery. Similar to our findings in genes of the γ-secretase complex, differential methylation of the promoter regions of genes involved in clathrin-mediated endocytosis (*CLTA, BIN1* and *CD2AP*) was also unique to neurons of the dentate gyrus. We cannot confidently predict the effect of promoter hypermethylation in *CLTA* and hypomethylation in *BIN1* and *CD2AP* on the expression of the respective proteins. Nevertheless, these results are interesting and support previous findings about the involvement of clathrin-associated endocytic proteins in Alzheimer’s disease [80–83]. Notably, these results could not be identified in the bulk methylation data (Fig. 5 and Additional File 1: Fig. S10), implying that cell type deconvolution can enable the discovery of novel molecular pathologic changes of AD. Our finding should motivate cell-type-specific re-analysis of previously published bulk methylation datasets to investigate what new insights into AD molecular pathology can be gleaned through the use of deconvolution. The lack of any clear association between bulk or CTS methylation and cognitive status is not surprising since major CTS methylation findings were related to Aβ, which is notoriously poorly correlated with clinical diagnosis of dementia.

### Limitations

Although we identified novel methylation differences in the oldest old that were associated with ADNC, our study had several limitations. We used the *Illumina 850k* platform, which only captures a small fraction of the human methylome and is not designed to capture DNA hydroxymethylation or other types of epigenetic changes. A general limitation of the *90* + *Study* cohort is the lack of ethnic diversity and the relatively higher education compared to the general oldest-old population in the US. Problems with DNA quality preserved in FFPE tissue further limited us in selecting a representative cohort, the cohort therefore did not reflect the overall distribution of ADNC and cognitive status of the full *90*+ *Study* cohort. While our cohort was uniquely old with a mean age of 97 (SD ± 3.5) years, we only had a limited sample size of 47 individuals, out of which only 4 individuals had low or no ADNC as measured by AD severity score (see Table 1). This possibly reduced our ability to replicate AD related methylation differences that were previously reported in several other studies [1, 2, 7, 8, 84]. Nevertheless, the discoveries we made in the DG were detectable despite the low sample size. Previous papers primarily described methylation differences in cerebral cortical regions (mainly frontal cortex) related to Braak stage for neurofibrillary degeneration, the strongest neuropathologic correlate of dementia. In our oldest old cohort, we did not have any individuals with low burden of neurofibrillary tangles as represented by an NIA-AA B score of 0 or 1 (similar to Braak stage II or less). Comparing individuals with NIA-AA B scores of 2 and 3, we did not detect any associations of methylation with neurofibrillary tangles in the frontal cortex, but detected some DMPTs in the dentate gyrus and cingulate gyrus. The cell type deconvolution approach used in our study allowed us to investigate cell-type-specific associations in bulk homogenate without the need to sort nuclei to obtain CTS profiles [10–12]. However, we were not able to recover CTS profiles for all cell types across every brain region, especially microglia, due to their relatively low estimated cell type proportions. This underscores the need to generate sorted CTS data for low abundance cell types, as recent studies have done for gene expression [85]. While we found several functionally relevant associations between altered DNA methylation and ADNC, future studies should validate our findings experimentally using sorted CTS data and high dimensional imaging. We also cannot determine whether the associations we found are causal or merely correlative, but they do provide interesting mechanistic hypotheses to test experimentally.

## Conclusion

We presented a novel dataset of bulk methylation at eight precisely dissected brain regions in the old-aged human brain. We applied computational deconvolution as a powerful method to recover cell-type-specific signals without the need for actual cell sorting. With a total of 47 cases, our unique old-aged cohort was small and skewed towards higher burdens of AD neuropathology. We were therefore not able to replicate findings from previous studies where differential methylation was related to Braak stages in the frontal cortex. Nevertheless, we discovered a high amount of biologically meaningful methylation differences related to AD neuropathology mainly in the dentate gyrus and cingulate gyrus. Both regions have previously been underrepresented in methylation studies of AD. In neurons of the dentate gyrus, increased Aβ plaque burden was associated with promoter hypomethylation of two important genes of the γ-secretase complex (*PEN-2, NCSTN*), a complex that is involved in the cleavage of amyloid precursor protein into Aβ. Our dataset was made publicly available and can serve as a brain region reference panel for future studies and help advance research in aging and neurodegenerative diseases.

## Supplementary Information

**Additional file 1**: This file contains all supplementary figures referred to in the manuscript.

**Additional file 2**: Statistics from correlation analysis of batch variables with neuropathological scores (kruskal wallis) and clinical diagnosis (G-test). Description: This table displays the results from the correlation analysis of batch variables with neuropathological scores and clinical diagnosis. For this analysis, Kruskal wallis and G-test were used respectively.

**Additional file 3**: Overview of datasets removed due to low regional dimensions. This table contains the numbers of detected regional dimensions for each methylation dataset. It accompanies the filtering due to low regional dimensions as described in the methods section.

**Additional file 4**: Case-level demographics and neuropathological scoring data. This file contains detailed demographics and neuropathological scoring data for each individual study participant.

**Additional file 5**: Statistics for all significant differentially methylated promoter regions (DMPTs). This file contains multiple tables with statistics for all significant differentially methylated promoter regions (DMPTs) detected through the analysis explained in this manuscript.

## Data Availability

The methylation datasets of 47 individuals generated during this study are available in the GEO repository under GSE212682, https://www.ncbi.nlm.nih.gov/geo/query/acc.cgi?acc=GSE212682. To ensure high data privacy standards, the demographics data available on GEO does not include information on the age of participants. All data analysis code is publicly available on github https://github.com/anna-lena-lang/90plus_brain_methylation.

## References

[1] Smith RG, Hannon E, De Jager PL (2018). Elevated DNA methylation across a 48-kb region spanning the HOXA gene cluster is associated with Alzheimer’s disease neuropathology. Alzheimers Dement.

[2] De Jager PL, Srivastava G, Lunnon K (2014). Alzheimer’s disease: early alterations in brain DNA methylation at ANK1, BIN1, RHBDF2 and other loci. Nat Neurosci.

[3] Lardenoije R, Roubroeks JAY, Pishva E (2019). Alzheimer’s disease-associated (hydroxy)methylomic changes in the brain and blood. Clin Epigenet.

[4] Beach TG, Adler CH, Sue LI (2015). Arizona study of aging and neurodegenerative disorders and brain and body donation program. Neuropathology.

[5] Gasparoni G, Bultmann S, Lutsik P (2018). DNA methylation analysis on purified neurons and glia dissects age and Alzheimer’s disease-specific changes in the human cortex. Epigen Chrom.

[6] Shireby GL, Davies JP, Francis PT (2020). Recalibrating the epigenetic clock: implications for assessing biological age in the human cortex. Brain.

[7] Lunnon K, Smith R, Hannon E (2014). Methylomic profiling implicates cortical deregulation of ANK1 in alzheimer’s disease. Nat Neurosci.

[8] Smith AR, Smith RG, Pishva E (2019). Parallel profiling of DNA methylation and hydroxymethylation highlights neuropathology-associated epigenetic variation in alzheimer’s disease. Clin Epigen.

[9] Shireby G, Dempster EL, Policicchio S (2022). DNA methylation signatures of alzheimer’s disease neuropathology in the cortex are primarily driven by variation in non-neuronal cell-types. Nat Commun.

[10] Titus AJ, Gallimore RM, Salas LA, Christensen BC (2017). Cell-type deconvolution from DNA methylation: a review of recent applications. Hum Mol Genet.

[11] Rahmani E, Schweiger R, Rhead B (2019). Cell-type-specific resolution epigenetics without the need for cell sorting or single-cell biology. Nat Commun.

[12] Zhu T, Liu J, Beck S (2022). A pan-tissue DNA methylation atlas enables in silico decomposition of human tissue methylomes at cell-type resolution. Nat Meth.

[13] Corrada MM, Berlau DJ, Kawas CH (2012). A population-based clinicopathological study in the oldest-old: the 90+ study. Curr Alzheimer Res.

[14] Rhein M, Hagemeier L, Klintschar M (2015). DNA methylation results depend on DNA integrity-role of post mortem interval. Front Genet.

[15] Montine TJ, Phelps CH, Beach TG (2012). National institute on aging-alzheimer’s association guidelines for the neuropathologic assessment of alzheimer's disease: a practical approach. Acta Neuropathol.

[16] Hyman BT, Phelps CH, Beach TG (2012). National Institute on Aging–Alzheimer’s Association guidelines for the neuropathologic assessment of Alzheimer’s disease. Alzheimers Dement.

[17] Mirra SS, Heyman A, McKeel D (1991). The Consortium to Establish a Registry for Alzheimer’s Disease (CERAD): Part II. Standardization of the neuropathologic assessment of Alzheimer's disease. Neurology.

[18] Kawas C, Segal J, Stewart WF (1994). A validation study of the Dementia Questionnaire. Arch Neurol.

[19] Clark CM, Ewbank DC (1996). Performance of the dementia severity rating scale: a caregiver questionnaire for rating severity in Alzheimer disease. Alzheimer Dis Assoc Disord.

[20] Pfeffer RI, Kurosaki TT, Harrah CH (1982). Measurement of functional activities in older adults in the community. J Gerontol.

[21] Folstein MF, Folstein SE, McHugh PR (1975). “Mini-mental state”: a practical method for grading the cognitive state of patients for the clinician. J Psychiatr Res.

[22] American Psychiatric Association Staff (2010) Diagnostic and Statistical Manual of Mental Disorders, Fourth Edition, Text Revision (DSM-IV-TR®). American Psychiatric Pub

[23] Corrada MM, Sonnen JA, Kim RC, Kawas CH (2016). Microinfarcts are common and strongly related to dementia in the oldest-old: The 90+ study. Alzheimers Dement.

[24] Quick-DNA FFPE Miniprep. In: ZYMO RESEARCH. https://www.zymoresearch.com/products/quick-dna-ffpe-miniprep. Accessed 16 Aug 2019

[25] Qubit dsDNA BR Assay Kit - Thermo Fisher Scientific. https://www.thermofisher.com/order/catalog/product/Q32850?SID=srch-srp-Q32850. Accessed 16 Aug 2019

[26] Fortin J-P, Triche TJ, Hansen KD (2017). Preprocessing, normalization and integration of the Illumina HumanMethylationEPIC array with minfi. Bioinformatics.

[27] Murat K, Grüning B, Poterlowicz PW (2020). Ewastools: infinium human methylation beadchip pipeline for population epigenetics integrated into galaxy. Gigascience.

[28] Aryee MJ, Jaffe AE, Corrada-Bravo H (2014). Minfi: a flexible and comprehensive bioconductor package for the analysis of Infinium DNA methylation microarrays. Bioinformatics.

[29] Pidsley R, Zotenko E, Peters TJ (2016). Critical evaluation of the Illumina MethylationEPIC BeadChip microarray for whole-genome DNA methylation profiling. Genome Biol.

[30] McCartney DL, Walker RM, Morris SW (2016). Identification of polymorphic and off-target probe binding sites on the Illumina Infinium MethylationEPIC BeadChip. Genom Data.

[31] Müller F, Scherer M, Assenov Y (2019). RnBeads 2.0: comprehensive analysis of DNA methylation data. Genome Biol.

[32] Teschendorff AE, Marabita F, Lechner M (2013). A beta-mixture quantile normalization method for correcting probe design bias in Illumina Infinium 450 k DNA methylation data. Bioinformatics.

[33] Tian Y, Morris TJ, Webster AP (2017). ChAMP: updated methylation analysis pipeline for Illumina BeadChips. Bioinformatics.

[34] Zhu T, Liu J, Beck S, Pan S, Capper D, Lechner M, Thirlwell C, Breeze CE, Teschendorff AE (2022) Github EPISCORE data repository. https://github.com/aet21/EpiSCORE. Accessed 27 Mar 2022

[35] GTEx Consortium (2020). The GTEx consortium atlas of genetic regulatory effects across human tissues. Science.

[36] Du P, Zhang X, Huang C-C (2010). Comparison of Beta-value and M-value methods for quantifying methylation levels by microarray analysis. BMC Bioinform.

[37] Marčenko VA, Pastur LA (1967). Distribution of eigenvalues for some sets of random matrices. Math USSR.

[38] Ritchie ME, Phipson B, Wu D (2015). limma powers differential expression analyses for RNA-sequencing and microarray studies. Nucleic Acids Res.

[39] Wickham H (2016). ggplot2: elegant graphics for data analysis.

[40] Durinck S, Spellman PT, Birney E, Huber W (2009). Mapping identifiers for the integration of genomic datasets with the R/Bioconductor package biomaRt. Nat Protoc.

[41] He W, Muenchrath MN (2011) 90+ in the United States: 2006–2008. US department of commerce, economics and statistics administration, US

[42] Watson CT, Roussos P, Garg P (2016). Genome-wide DNA methylation profiling in the superior temporal gyrus reveals epigenetic signatures associated with Alzheimer’s disease. Genome Med.

[43] Semick SA, Bharadwaj RA, Collado-Torres L (2019). Integrated DNA methylation and gene expression profiling across multiple brain regions implicate novel genes in Alzheimer’s disease. Acta Neuropathol.

[44] Andrade-Moraes CH, Oliveira-Pinto AV, Castro-Fonseca E (2013). Cell number changes in Alzheimer’s disease relate to dementia, not to plaques and tangles. Brain.

[45] Guintivano J, Aryee MJ, Kaminsky ZA (2013). A cell epigenotype specific model for the correction of brain cellular heterogeneity bias and its application to age, brain region and major depression. Epigenetics.

[46] Illingworth RS, Gruenewald-Schneider U, De Sousa D (2015). Inter-individual variability contrasts with regional homogeneity in the human brain DNA methylome. Nucleic Acids Res.

[47] Rizzardi LF, Hickey PF, Rodriguez DiBlasi V (2019). Neuronal brain-region-specific DNA methylation and chromatin accessibility are associated with neuropsychiatric trait heritability. Nat Neurosci.

[48] Mittelbronn M, Dietz K, Schluesener HJ, Meyermann R (2001). Local distribution of microglia in the normal adult human central nervous system differs by up to one order of magnitude. Acta Neuropathol.

[49] Valério-Gomes B, Guimarães DM, Szczupak D, Lent R (2018). The absolute number of oligodendrocytes in the adult mouse brain. Front Neuroanat.

[50] von Bartheld CS, Bahney J, Herculano-Houzel S (2016). The search for true numbers of neurons and glial cells in the human brain: a review of 150 years of cell counting. J Comp Neurol.

[51] McInnes L, Healy J, Melville J (2018) UMAP: uniform manifold approximation and projection for dimension reduction. arXiv [stat.ML]

[52] Corces MR, Shcherbina A, Kundu S (2020). Single-cell epigenomic analyses implicate candidate causal variants at inherited risk loci for alzheimer’s and parkinson's diseases. Nat Genet.

[53] Bellenguez C, Küçükali F, Jansen IE (2022). New insights into the genetic etiology of Alzheimer’s disease and related dementias. Nat Genet.

[54] Chouliaras L, Mastroeni D, Delvaux E (2013). Consistent decrease in global DNA methylation and hydroxymethylation in the hippocampus of Alzheimer’s disease patients. Neurobiol Aging.

[55] Jia Y, Wang N, Zhang Y (2020). Alteration in the function and expression of SLC and ABC transporters in the neurovascular unit in Alzheimer’s disease and the clinical significance. Aging Dis.

[56] Cristóvão JS, Gomes CM (2019). S100 proteins in Alzheimer’s disease. Front Neurosci.

[57] Wolfe MS (2003). Gamma-se cretase–intramembrane protease with a complex. Sci Aging Knowl Environ.

[58] André P, Samieri C, Buisson C (2019). Lipopolysaccharide-binding protein, soluble CD14, and the long-term risk of alzheimer’s disease: a nested case-control pilot study of older community dwellers from the three-city cohort. J Alzheimers Dis.

[59] Baylin SB (2005). DNA methylation and gene silencing in cancer. Nat Clin Pract Oncol.

[60] Ball MP, Li JB, Gao Y (2009). Targeted and genome-scale strategies reveal gene-body methylation signatures in human cells. Nat Biotechnol.

[61] Kang JG, Park JS, Ko J-H, Kim Y-S (2019). Regulation of gene expression by altered promoter methylation using a CRISPR/Cas9-mediated epigenetic editing system. Sci Rep.

[62] De Strooper B (2003). Aph-1, Pen-2, and Nicastrin with Presenilin generate an active gamma-Secretase complex. Neuron.

[63] Hur J-Y (2022). γ-secretase in alzheimer’s disease. Exp Mol Med.

[64] Luo W-J, Wang H, Li H (2003). PEN-2 and APH-1 coordinately regulate proteolytic processing of presenilin 1. J Biol Chem.

[65] Takasugi N, Tomita T, Hayashi I (2003). The role of presenilin cofactors in the gamma-secretase complex. Nature.

[66] Lanoiselée H-M, Nicolas G, Wallon D (2017). APP, PSEN1, and PSEN2 mutations in early-onset Alzheimer disease: a genetic screening study of familial and sporadic cases. PLoS Med.

[67] Libard S, Cerjan D, Alafuzoff I (2019). Characteristics of the tissue section that influence the staining outcome in immunohistochemistry. Histochem Cell Biol.

[68] Chu Y, Peng X, Long Z (2015). Distribution and expression of Pen-2 in the central nervous system of APP/PS1 double transgenic mice. Acta Biochim Biophys Sin.

[69] Brodsky FM (2012). Diversity of clathrin function: new tricks for an old protein. Annu Rev Cell Dev Biol.

[70] Thomas RS, Lelos MJ, Good MA, Kidd EJ (2011). Clathrin-mediated endocytic proteins are upregulated in the cortex of the Tg2576 mouse model of Alzheimer’s disease-like amyloid pathology. Biochem Biophys Res Commun.

[71] Cirrito JR, Kang J-E, Lee J (2008). Endocytosis is required for synaptic activity-dependent release of amyloid-beta in vivo. Neuron.

[72] Ando K, Brion J-P, Stygelbout V (2013). Clathrin adaptor CALM/PICALM is associated with neurofibrillary tangles and is cleaved in Alzheimer’s brains. Acta Neuropathol.

[73] Lambert JC, Ibrahim-Verbaas CA, Harold D (2013). Meta-analysis of 74,046 individuals identifies 11 new susceptibility loci for Alzheimer’s disease. Nat Genet.

[74] Harold D, Abraham R, Hollingworth P (2009). Genome-wide association study identifies variants at CLU and PICALM associated with Alzheimer’s disease. Nat Genet.

[75] Smith RG, Pishva E, Shireby G (2021). A meta-analysis of epigenome-wide association studies in Alzheimer’s disease highlights novel differentially methylated loci across cortex. Nat Commun.

[76] Altuna M, Urdánoz-Casado A, Sánchez-Ruiz de Gordoa J (2019). DNA methylation signature of human hippocampus in Alzheimer’s disease is linked to neurogenesis. Clin Epigenetics.

[77] Sala Frigerio C, Piscopo P, Calabrese E (2005). PEN-2 gene mutation in a familial Alzheimer’s disease case. J Neurol.

[78] Campbell WA, Yang H, Zetterberg H (2006). Zebrafish lacking Alzheimer presenilin enhancer 2 (Pen-2) demonstrate excessive p53-dependent apoptosis and neuronal loss. J Neurochem.

[79] Bi H-R, Zhou C-H, Zhang Y-Z (2021). Neuron-specific deletion of presenilin enhancer2 causes progressive astrogliosis and age-related neurodegeneration in the cortex independent of the Notch signaling. CNS Neurosci Ther.

[80] Alsaqati M, Thomas RS, Kidd EJ (2018). Proteins involved in endocytosis are upregulated by ageing in the normal human brain: implications for the development of Alzheimer’s disease. J Gerontol A Biol Sci Med Sci.

[81] Lambert E, Saha O, Soares Landeira B (2022). The Alzheimer susceptibility gene BIN1 induces isoform-dependent neurotoxicity through early endosome defects. Acta Neuropathol Commun.

[82] McMahon HT, Boucrot E (2011). Molecular mechanism and physiological functions of clathrin-mediated endocytosis. Nat Rev Mol Cell Biol.

[83] Chapuis J, Hansmannel F, Gistelinck M (2013). Increased expression of BIN1 mediates Alzheimer genetic risk by modulating tau pathology. Mol Psychiatry.

[84] Brokaw DL, Piras IS, Mastroeni D (2020). Cell death and survival pathways in Alzheimer’s disease: an integrative hypothesis testing approach utilizing -omic data sets. Neurobiol Aging.

[85] de Paiva-Lopes K, Snijders GJL, Humphrey J (2022). Genetic analysis of the human microglial transcriptome across brain regions, aging and disease pathologies. Nat Genet.

