# Supplementary material for: Methylation differences in Alzheimer’s disease neuropathologic change in the aged human brain
Source: Acta Neuropathol Commun. 2022 Nov 29;10:174. doi: 10.1186/s40478-022-01470-0 (PMC9710143; doi:10.1186/s40478-022-01470-0)
Supplement: Supplementary file 1 — Additional file 1: This file contains all supplementary figures referred to in the manuscript. [file 40478_2022_1470_MOESM1_ESM.docx]

## **Additional File 1**

**Content:** Supplementary Figures

**Publication title:** Methylation differences in Alzheimer’s disease neuropathologic change in the aged human brain

**Journal:** Acta Neuropathologica Communications

**Authors**: Anna-Lena Lang, Tiffany Eulalio ,Eddie Fox,Koya Yakabi, Syed A Bukhari, Claudia H Kawas, Maria M Corrada, Stephen B Montgomery, Frank Heppner, David Capper, Daniel Nachun, Thomas J Montine

Corresponding author: Anna-Lena Lang, email: [annalena.lang.26@gmail.com](mailto:annalena.lang.26@gmail.com), phone: +4915756025551


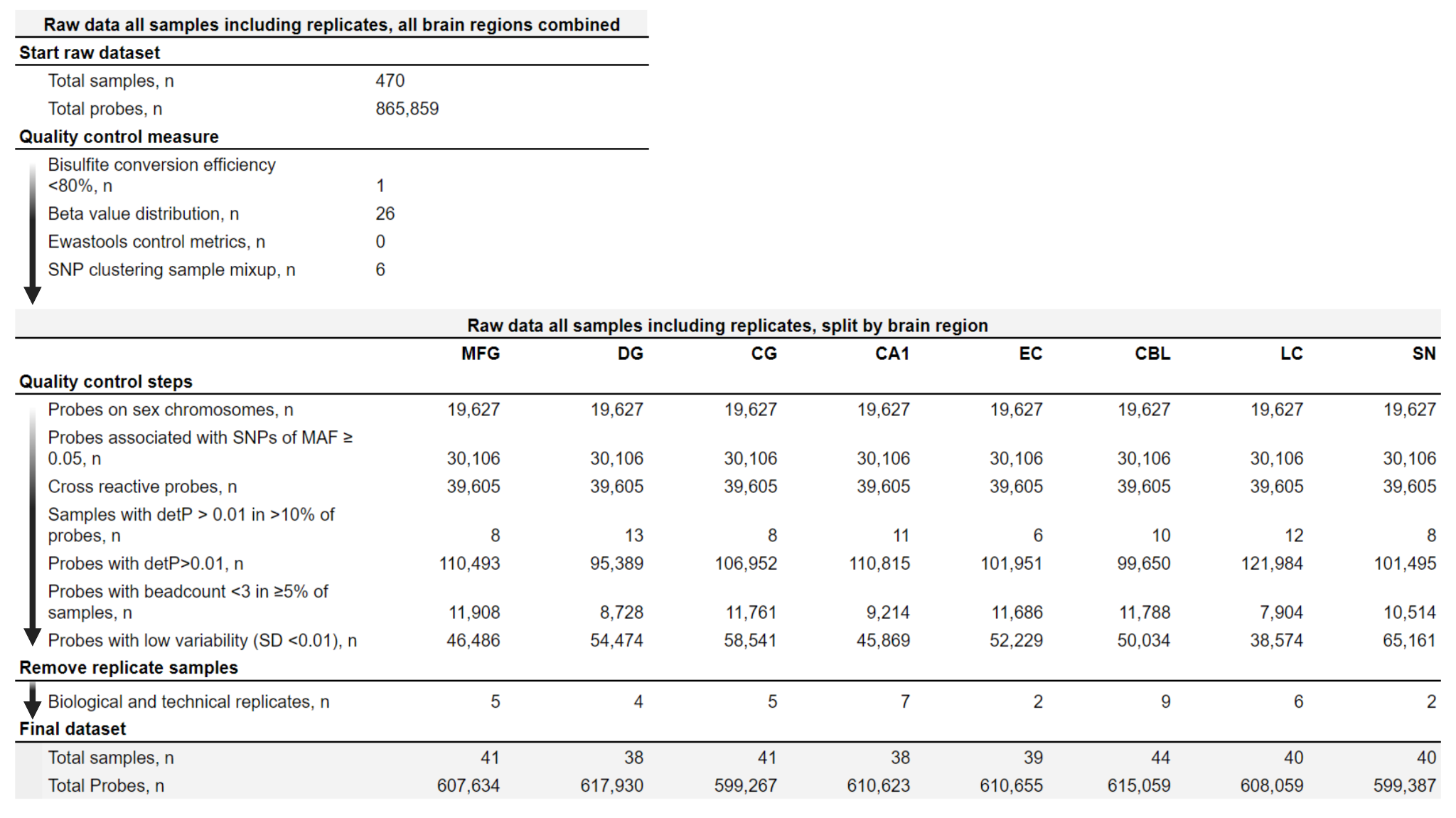


### **Supplementary Figure 1: Flowchart data filtering and quality control**

Flowchart visualizing the data filtering process. Abbreviations: *SNP* Single Nucleotide Polymorphism, *MAF* Minor Allele Frequency*, detP* detection p-value, *SD* Standard deviation, *Olig/OPCs* Oligodendrocytes/Oligodendrocyte Precursor Cells, *MFG* Middle Frontal Gyrus, *CG* Cingulate Gyrus, *DG* Dentate Gyrus, *EC* Entorhinal cortex, *SN* Substantia nigra, *LC*  Locus coeruleus, *CBM* Cerebellar cortex.

###

###
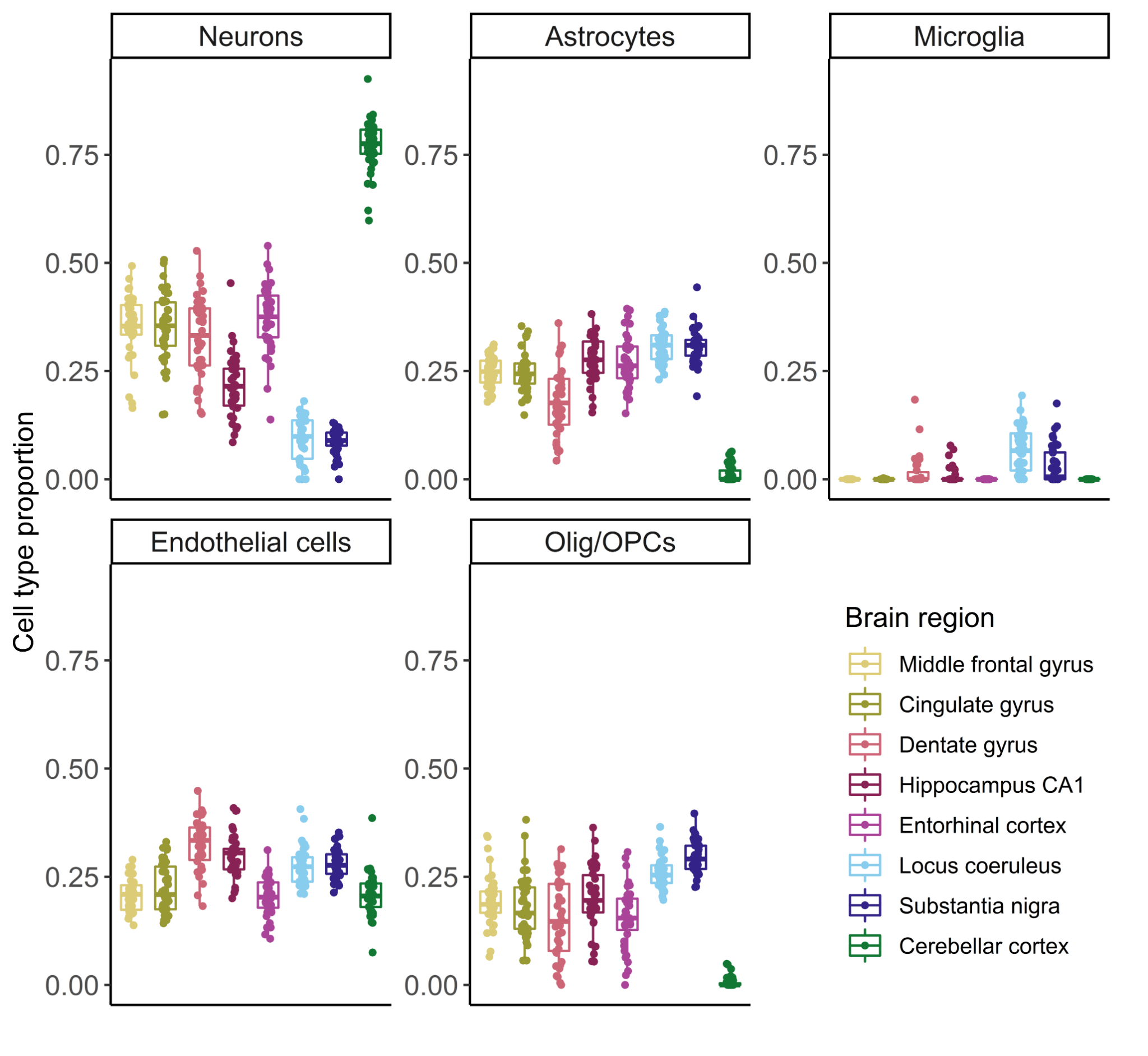
**Supplementary Figure 2: Overview of estimated cell type proportions from EpiSCORE by brain region**

Boxplots visualizing the predicted cell type proportions as derived from EpiSCORE analysis. Each figure shows the proportions of one cell type (Neurons, Astrocytes, Microglia, Endothelial cells, Oligodendrocytes/OPCs), with each boxplot displaying the cell type proportions for samples of the same brain region. Color coding reflects the different brain regions. Each dot represents one sample. The y-axis shows the estimated cell type proportion from 0 to 1. Abbreviations: *Olig/OPCs* Oligodendrocytes/Oligodendrocyte Precursor Cells.

###
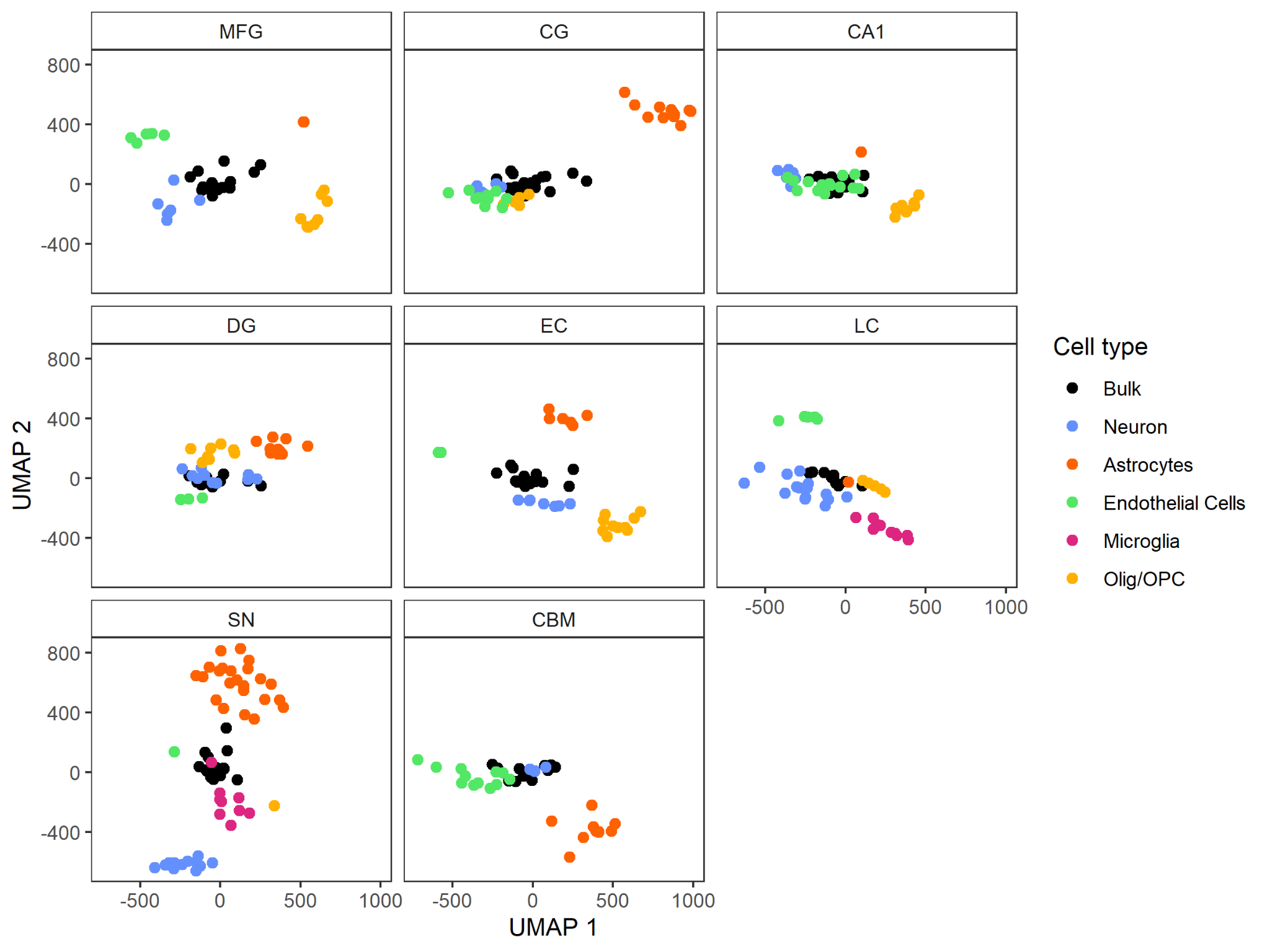


### Supplementary Figure 3: UMAP plot including bulk displaying clustering of brain region and cell type specific methylation data

We used the Uniform Manifold Approximation and Projection (UMAP) technique for dimension reduction to visualize similarities across cell type and brain region specific methylation data. Dimensionality reduction is performed with all brain regions and cell types combined. Each umap displays data from one brain region, axes are aligned across all brain regions. Each dot represents one individual sample. Colors reflect the cell types. Abbreviations: *Olig* Oligodendrocytes, *OPC* Oligodendrocyte Precursor Cells, *MFG* Middle Frontal Gyrus, *CG* Cingulate Gyrus, *DG* Dentate Gyrus, *EC* Entorhinal cortex, *SN* Substantia nigra, *LC*  Locus coeruleus, *CBM* Cerebellar cortex.


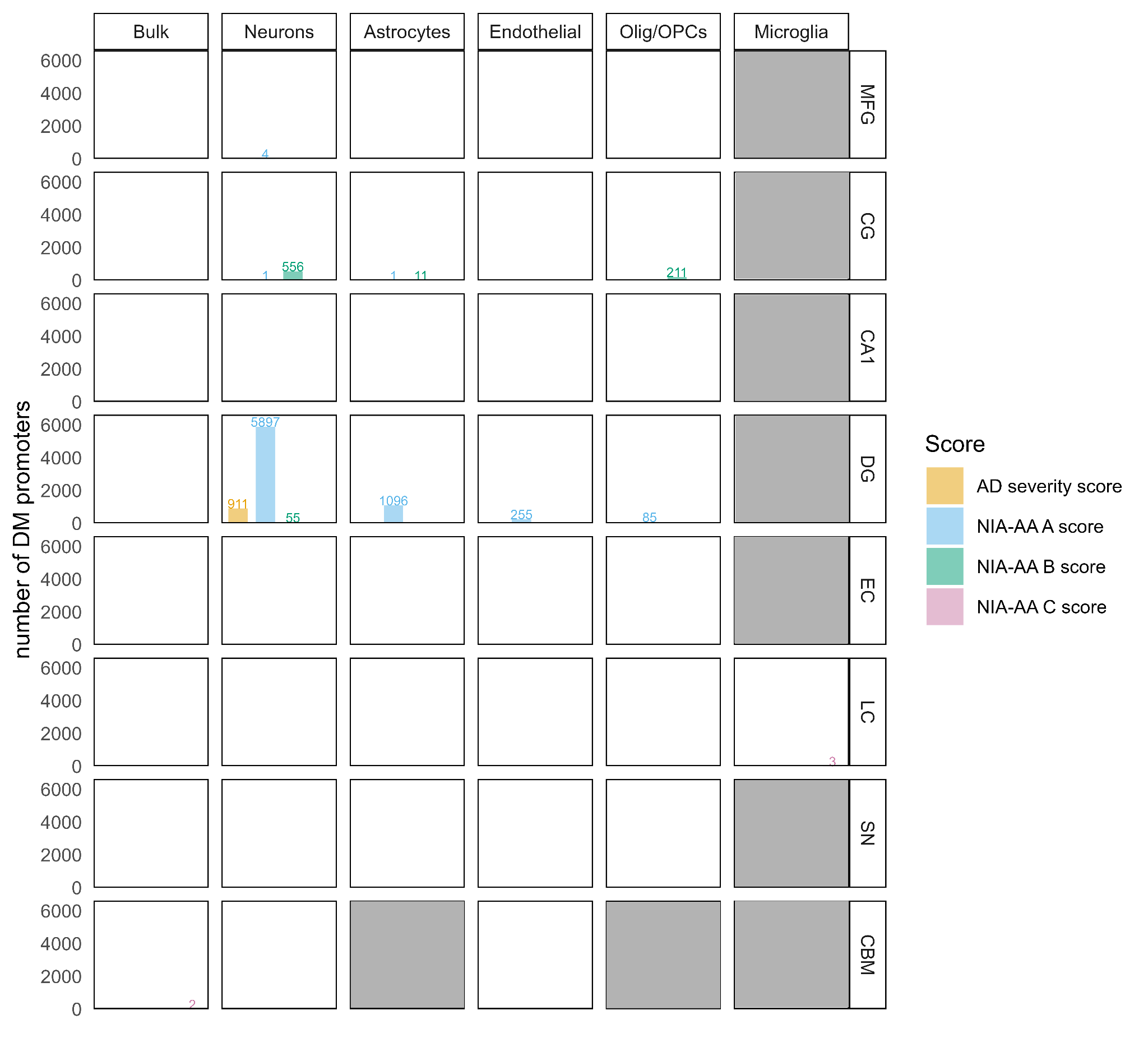


### **Supplementary Figure** 4**: Overview of brain region and cell type specific differentially methylated promoter regions of protein coding genes across different neuropathological scores**

Barplots display the number of significant (FDR < 0.05) differentially methylated promoter regions of protein coding genes. The plot is split by cell type as columns and brain regions as rows. Color coding of the bars reflects the different neuropathological scores. Comparisons shaded in gray were not analyzed due to weak cell type specific methylation signals within the given brain region (see Methods). Differential methylation of neurons was mainly detected in the Dentate Gyrus (DG) and Cingulate Gyrus (CG). In neurons in the dentate gyrus, 5897 differentially methylated promoters were found for the NIA-AA A score, 911 for the AD severity score and 55 for the B score. Those differences were not detectable in bulk data. Abbreviations*:* *DM* Differentially methylated, *Endothelial* Endothelial cells, *Olig/OPCs* Oligodendrocytes/Oligodendrocyte Precursor Cells, *MFG* Middle Frontal Gyrus, *CG* Cingulate Gyrus, *DG* Dentate Gyrus, *EC* Entorhinal cortex, *SN* Substantia nigra, *LC*  Locus coeruleus, *CBM* Cerebellar cortex, *NIA-AA* National institute of Aging Alzheimer's Association.


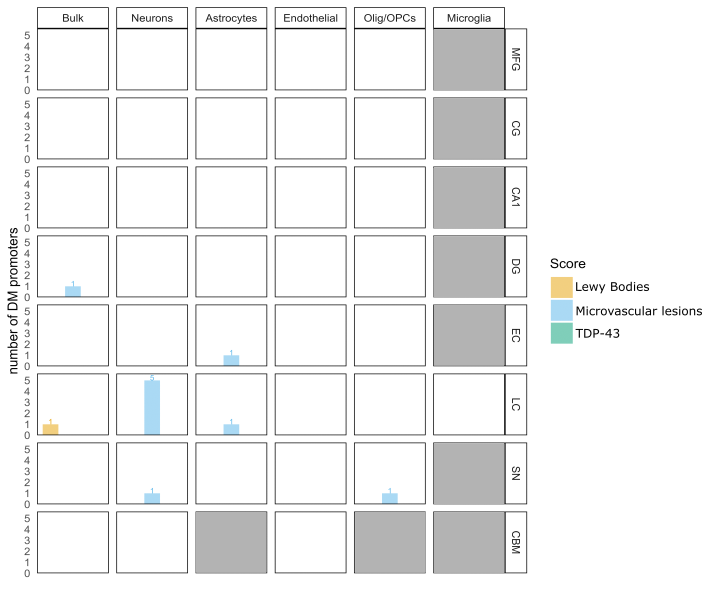


### Supplementary Figure 5: Overview of brain region and cell type specific differentially methylated promoter regions of protein coding genes across copathologies

Barplots display the number of significant (FDR < 0.05) differentially methylated promoter regions of protein coding genes. The plot is split by cell type as columns and brain regions as rows. Color coding of the bars reflects the different neuropathological scores of the three copathologies: Lewy bodies, microvascular lesions and TDP-43. Comparisons shaded in gray were not analyzed due to weak cell type specific methylation signals within the given brain region (see Methods). Abbreviations: *DM* Differentially methylated, *Endothelial* Endothelial cells, *Olig/OPCs* Oligodendrocytes/Oligodendrocyte Precursor Cells, *MFG* Middle Frontal Gyrus, *CG* Cingulate Gyrus, *DG* Dentate Gyrus, *EC* Entorhinal cortex, *SN* Substantia nigra, *LC*  Locus coeruleus, *CBM* Cerebellar cortex.


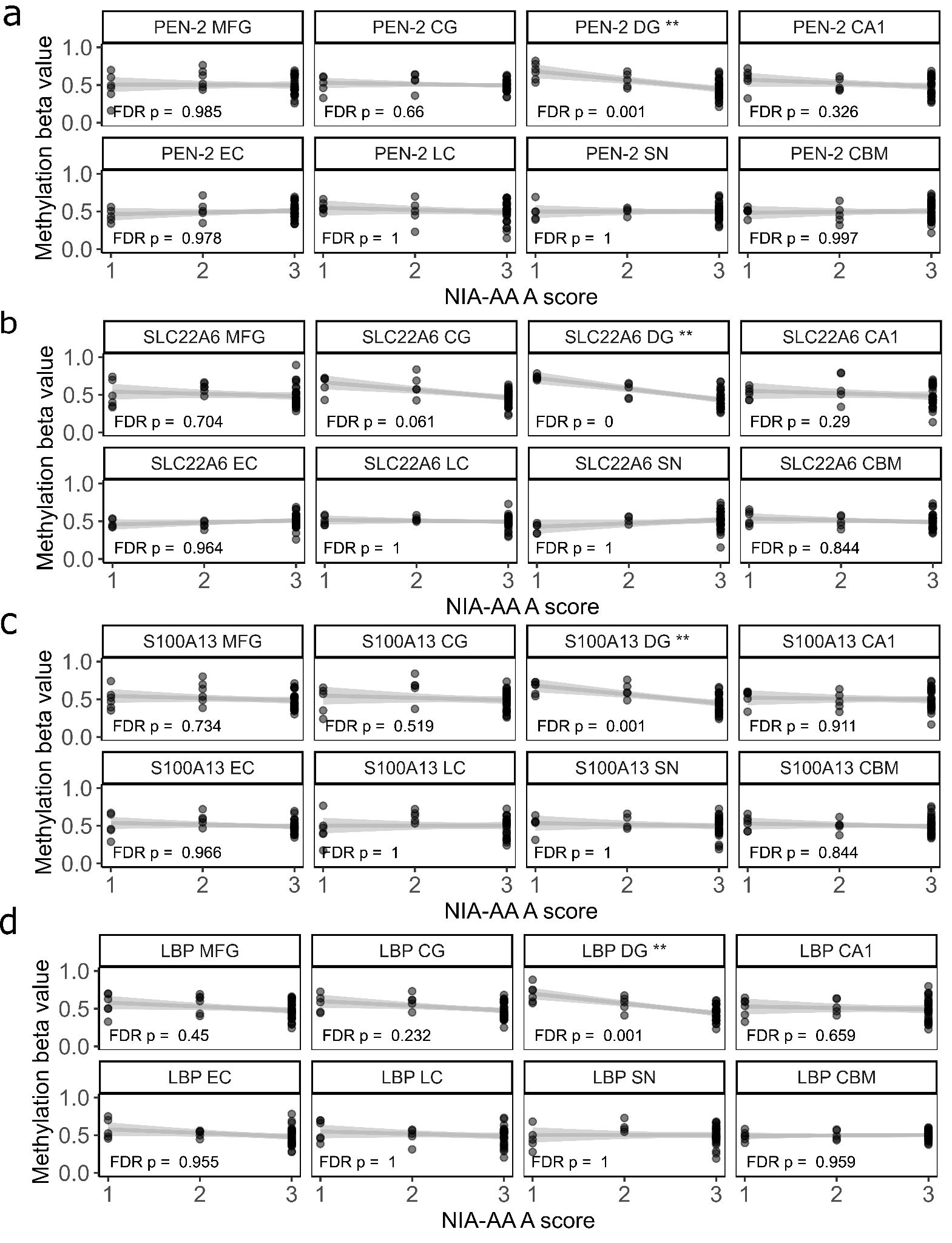


### **Supplementary Figure** 6**: Brain region specific neuronal methylation in known AD genes across individuals with different NIA-AA A scores**

Scatterplots with smoothers showing the relationship between neuronal methylation of the a) *PEN-2*, b) *SLC22A6*, c) *LBP* and d) *S100A13* gene across eight different brain regions (y-axis) from individuals with different NIA-AA A scores (x-axis). Methylation beta values are displayed on the y-axis and the categories of the NIA-AA A score on the x-axis. Each individual plot shows neuronal data from one brain region only. Each dot represents one individual sample. The standard linear regression was plotted as smoothers on top of the data: Smoothers curves are showing the relationship (solid line) between the NIA-AA A score and the methylation beta value. Shaded areas indicate the 95% confidence interval of the smooth curve. Out of all eight brain regions, the Dentate gyrus (DG) is the only region showing significant hypomethylation in these genes in neurons with increasing NIA-AA A score. **FDR p < 0.001. Abbreviations: *NIA-AA* National institute of Aging Alzheimer's Association, *AD* Alzheimer’s Disease.


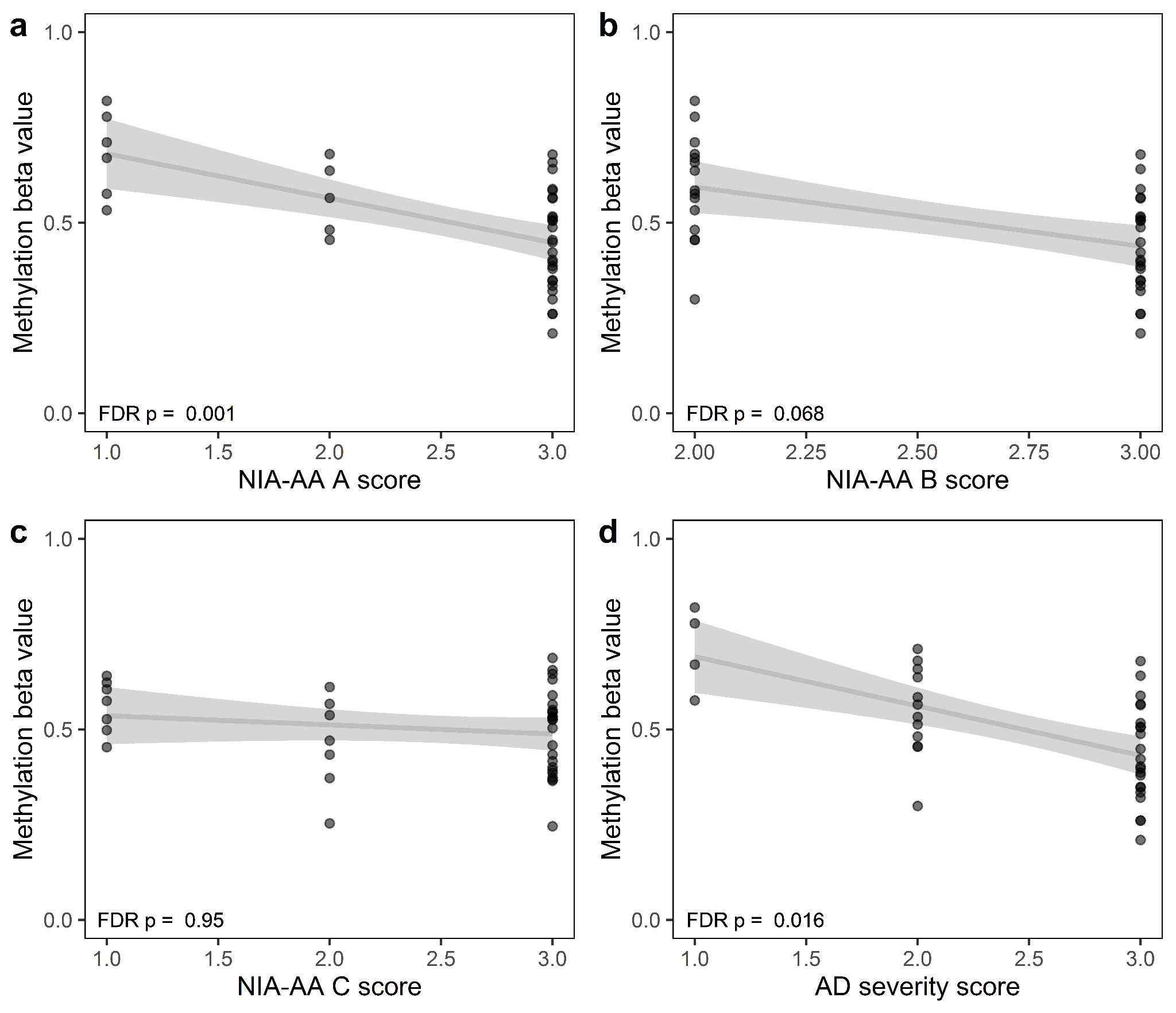


### **Supplementary Figure** 7**: *PEN-2* promoter methylation in neurons of the dentate gyrus across different neuropathological scores**

Scatterplots with smoothers showing the relationship between neuronal methylation of the promoter region of *PEN-2* (Presenilin enhancer 2) in the dentate gyrus from individuals with different neuropathological scores (x-axis). Methylation beta values are displayed on the y-axis. Each individual plot shows neuronal data for one neuropathological score. Each dot represents one individual sample. The standard linear regression was plotted as smoothers on top of the data: Smoothers curves are showing the relationship (solid line) between the neuropathological score and the methylation beta value. Shaded areas indicate the 95% confidence interval of the smooth curve. *PEN-2* shows significant hypomethylation with increasing NIA-AA A score (A) and Alzheimer’s disease severity score (D). FDR p-values are displayed in the lower left corner of the plots. Abbreviations: *DG* dentate gyrus, *PEN-2* presenilin enhancer 2, *NIA-AA* National institute of Aging Alzheimer's Association, *AD* Alzheimer’s Disease.


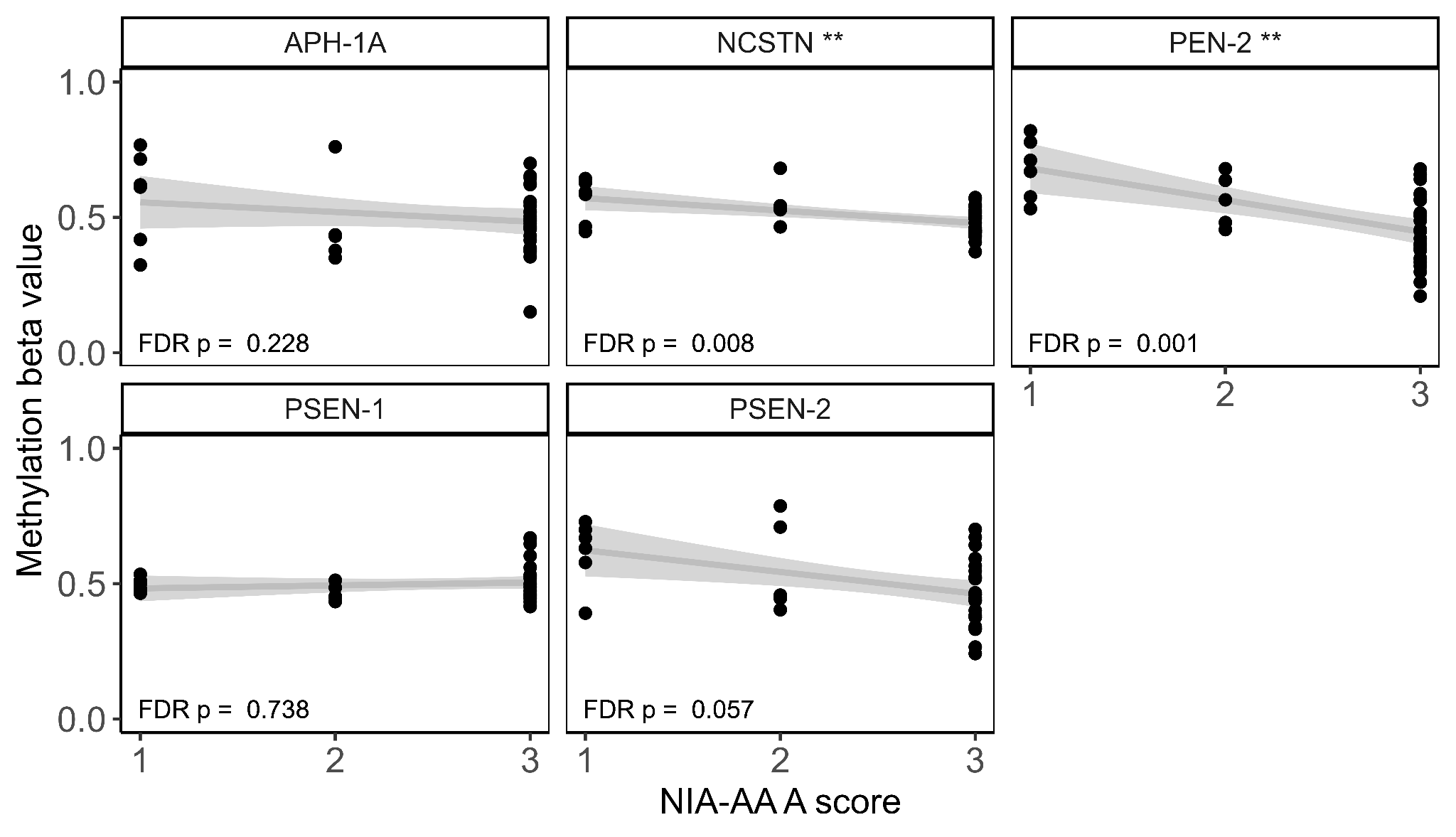


### **Supplementary Figure** 8**: Neuronal methylation in the dentate gyrus by amyloid burden in genes of the gamma-secretase complex**

Scatterplots with smoothers showing the relationship between neuronal methylation of promoter regions of genes of the gamma-secretase complex in the dentate gyrus from individuals with different NIA-AA A scores (x-axis). Methylation beta values are displayed on the y-axis and the categories of the NIA-AA A score on the x-axis. Each individual plot shows neuronal data for one promoter region. Each dot represents one individual sample. The standard linear regression was plotted as smoothers on top of the data: Smoothers curves are showing the relationship (solid line) between the NIA-AA A score and the methylation beta value. Shaded areas indicate the 95% confidence interval of the smooth curve. Out of all five genes, *NCSTN* and *PEN-2* show significant hypomethylation with increasing NIA-AA A score. FDR adjusted p-values are displayed in the plot. Abbreviations: *NCSTN* nicastrin, *PEN-2* presenilin enhancer 2, *PSEN-1* presenilin 1, *PSEN-2* presenilin 2, *APH-1A* aph-1 homolog A.


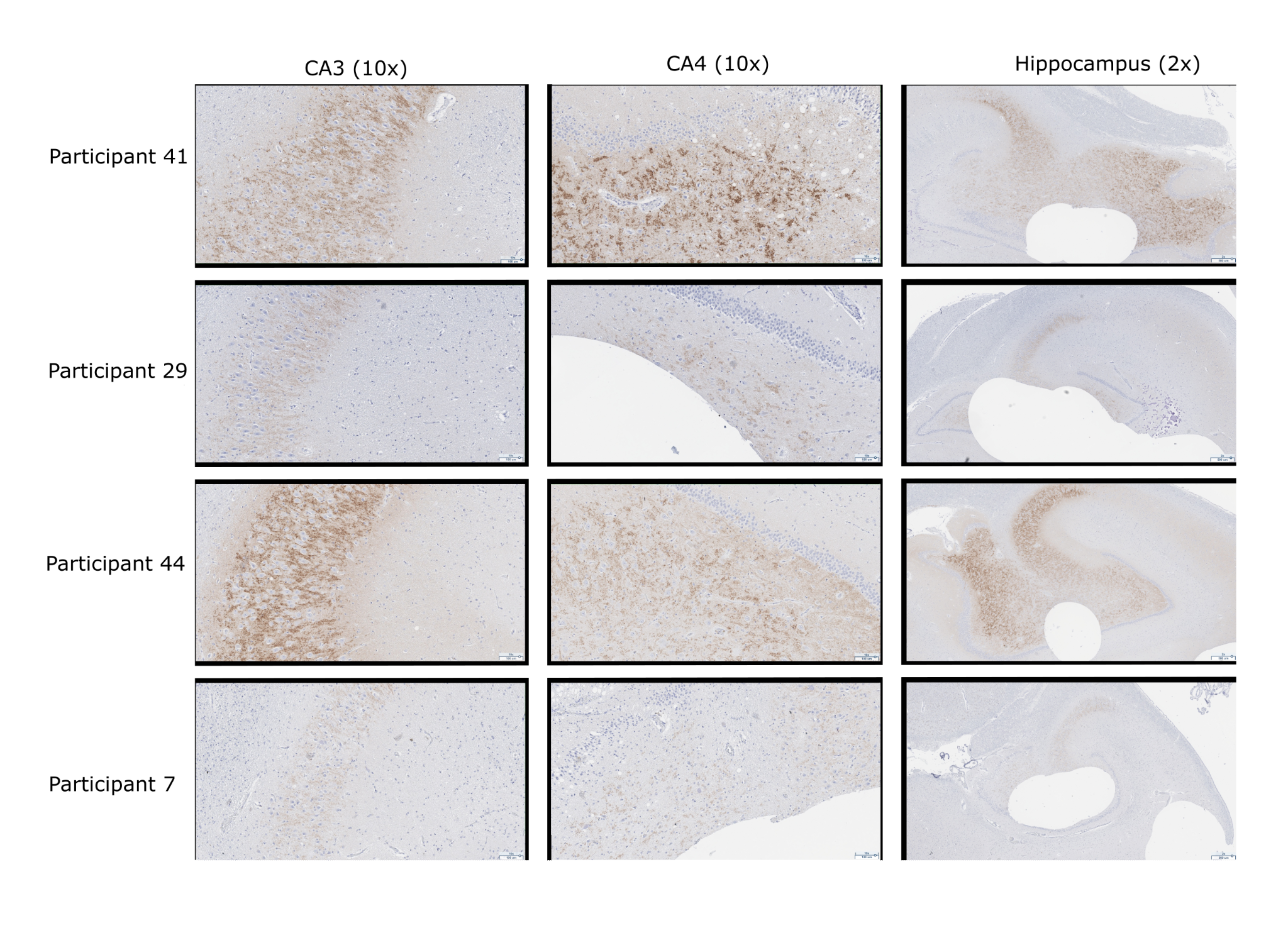


### **Supplementary Figure** 9**: Immunohistochemistry staining of *PEN-2* in hippocampus of individuals with different burden of Alzheimer’s Disease neuropathological change**

Figure showing Immunohistochemistry staining of *PEN-2* in the hippocampus of four individuals. Sample-IDs are displayed on the left. *Participants 41* and *29* both have low or no Alzheimer’s Disease neuropathologic changes (ADNC) as measured by Alzheimer’s Disease Severity Score (see *Online Resource 4* for extended phenotype data of all samples). *Participants 44* and *7* both have high ADNC. Abbreviations: *CA3 =* hippocampal region Cornu Ammonis 3, *CA4* = hippocampal region Cornu Ammonis 4

###
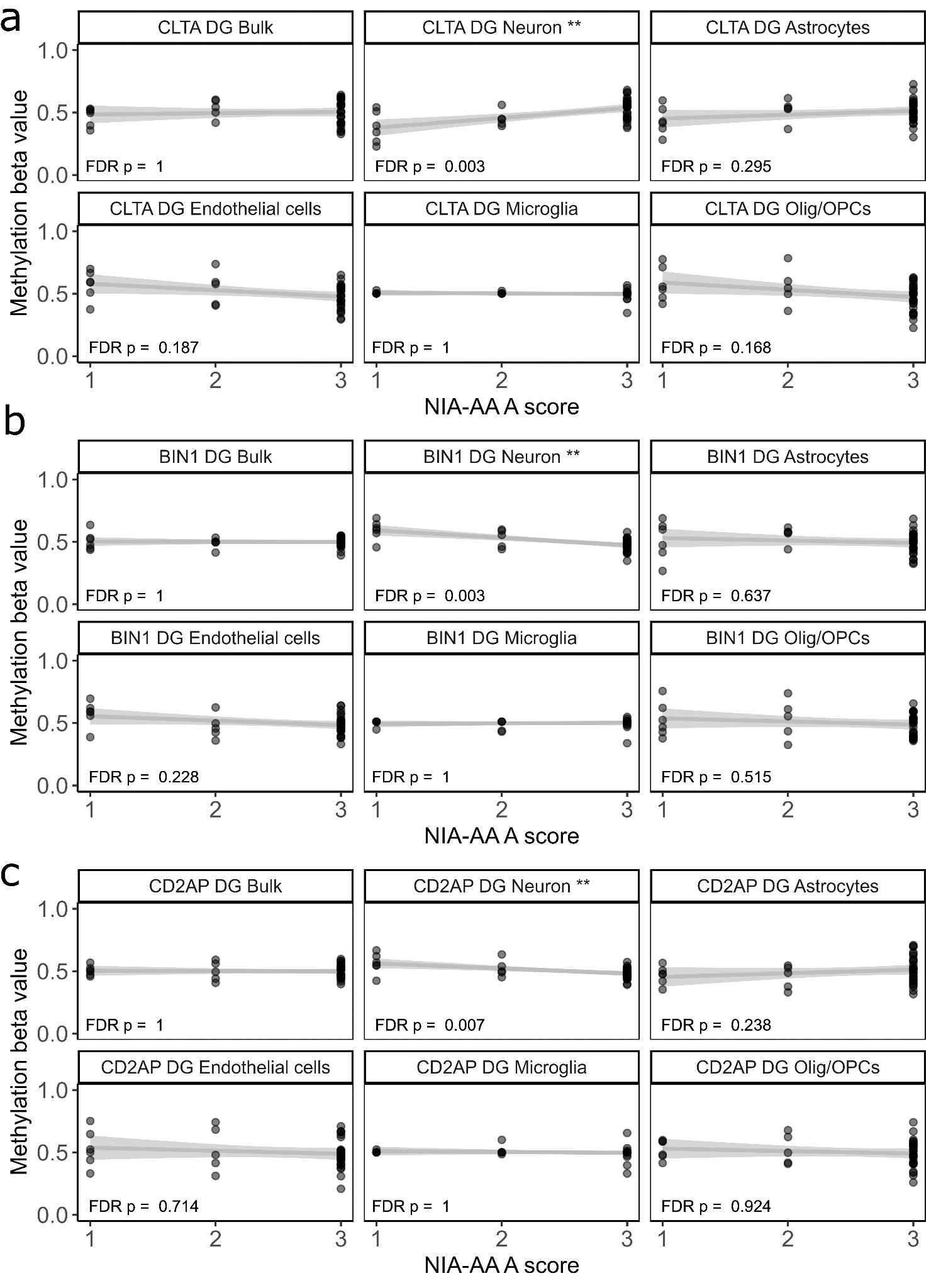
Supplementary Figure 10: Cell-type-specific promoter methylation of the CLTA, BIN1 and CD2AP gene in neurons of the dentate gyrus (DG) across individuals with different NIA-AA A scores

Scatterplots with smoothers showing the relationship between neuronal methylation of the promoter region of the a) CLTA (Clathrin Light Chain A) gene, b) *BIN1* (Bridging Integrator 1) gene and c) *CD2AP* (CD2 Associated Protein) gene (y-axis) across the five different cell types and bulk data from individuals with different Aβ plaque burden (NIA-AA A scores, x-axis). Methylation beta values are displayed on the y-axis and the categories of the NIA-AA A score on the x-axis. Each individual plot shows data from the dentate gyrus (DG) for different cell types. Each dot represents one individual sample. The standard linear regression was plotted as smoothers on top of the data: Smoothers curves are showing the relationship (solid line) between the NIA-AA A score and the methylation beta-value. Shaded areas indicate the 95% confidence interval of the smooth curve. We saw significant hypermethylation in the promoter region of the *CLTA* gene with increasing Aβ plaque burden only in neurons of the dentate gyrus and hypomethylation in *BIN1* and *CD2AP*. **FDR p < 0.01. *Olig/OPCs* Oligodendrocytes/Oligodendrocyte Precursor Cells. *DG* Dentate gyrus, *Aβ* Amyloid beta.


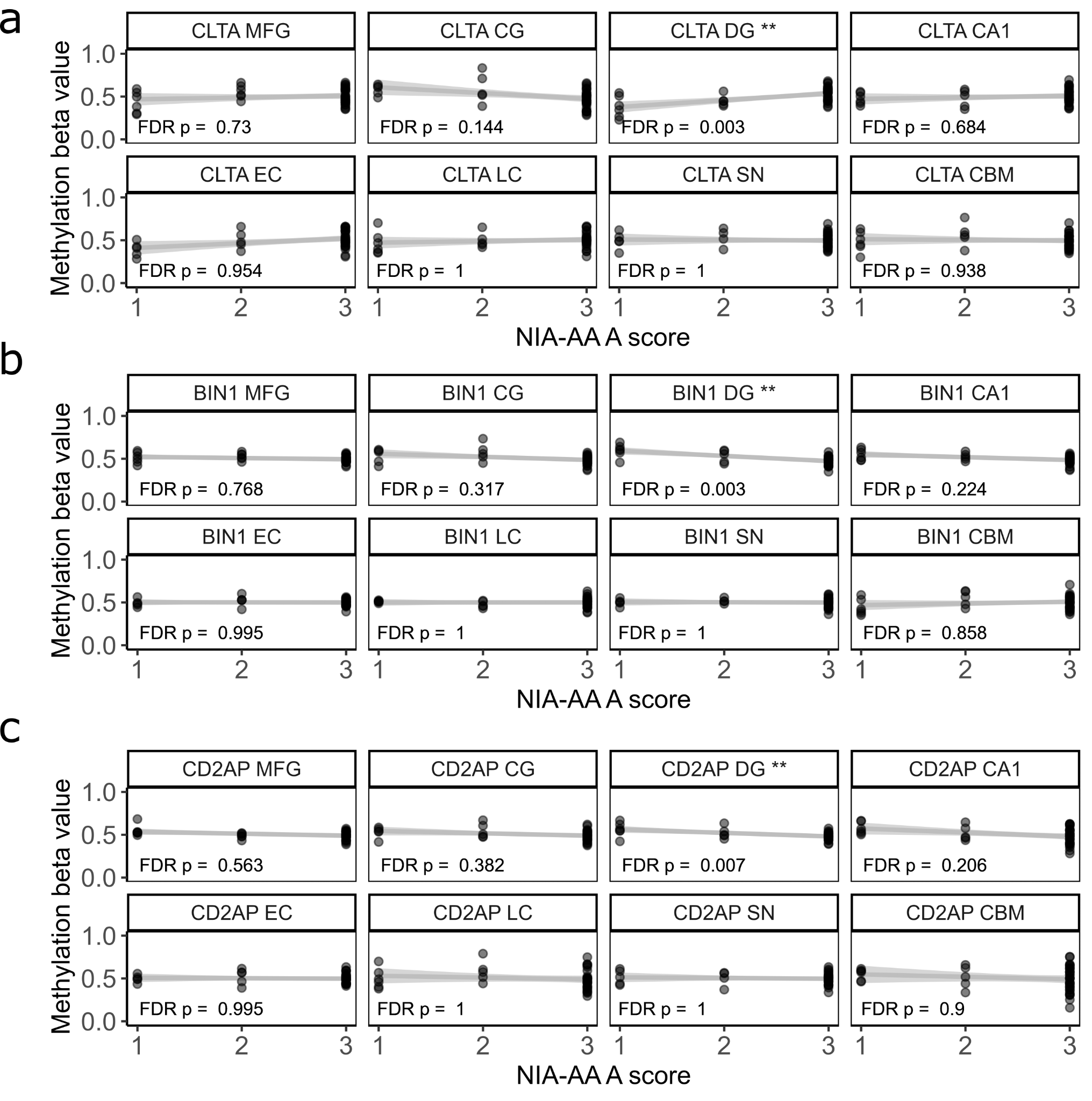


### Supplementary Figure 11: Brain region specific neuronal methylation in *CLTA, BIN1* and *C2AP* gene across individuals with different NIA-AA A scores

Scatterplots with smoothers showing the relationship between neuronal methylation of a) the *CLTA* (Clathrin Light Chain A) gene, b) *BIN1* (Bridging Integrator 1) gene and c) *CD2AP* (CD2 Associated Protein) gene across eight different brain regions (y-axis) from individuals with different NIA-AA A scores (x-axis). Methylation beta values are displayed on the y-axis and the categories of the NIA-AA A score on the x-axis. Each individual plot shows neuronal data from one brain region only. Each dot represents one individual sample. The standard linear regression was plotted as smoothers on top of the data: Smoothers curves are showing the relationship (solid line) between the NIA-AA A score and the methylation beta value. Shaded areas indicate the 95% confidence interval of the smooth curve. Out of all eight brain regions, the Dentate gyrus (DG) is the only region showing significant hypermethylation in these genes in neurons with increasing NIA-AA A score. **FDR p < 0.01, logFC = 0.50. Abbreviations: *NIA-AA* National institute of Aging Alzheimer's Association, *AD* Alzheimer’s Disease, *MFG* Middle Frontal Gyrus, *CG* Cingulate Gyrus, *DG* Dentate Gyrus, *EC* Entorhinal cortex, *SN* Substantia nigra, *LC*  Locus coeruleus, *CBM* Cerebellar cortex, .


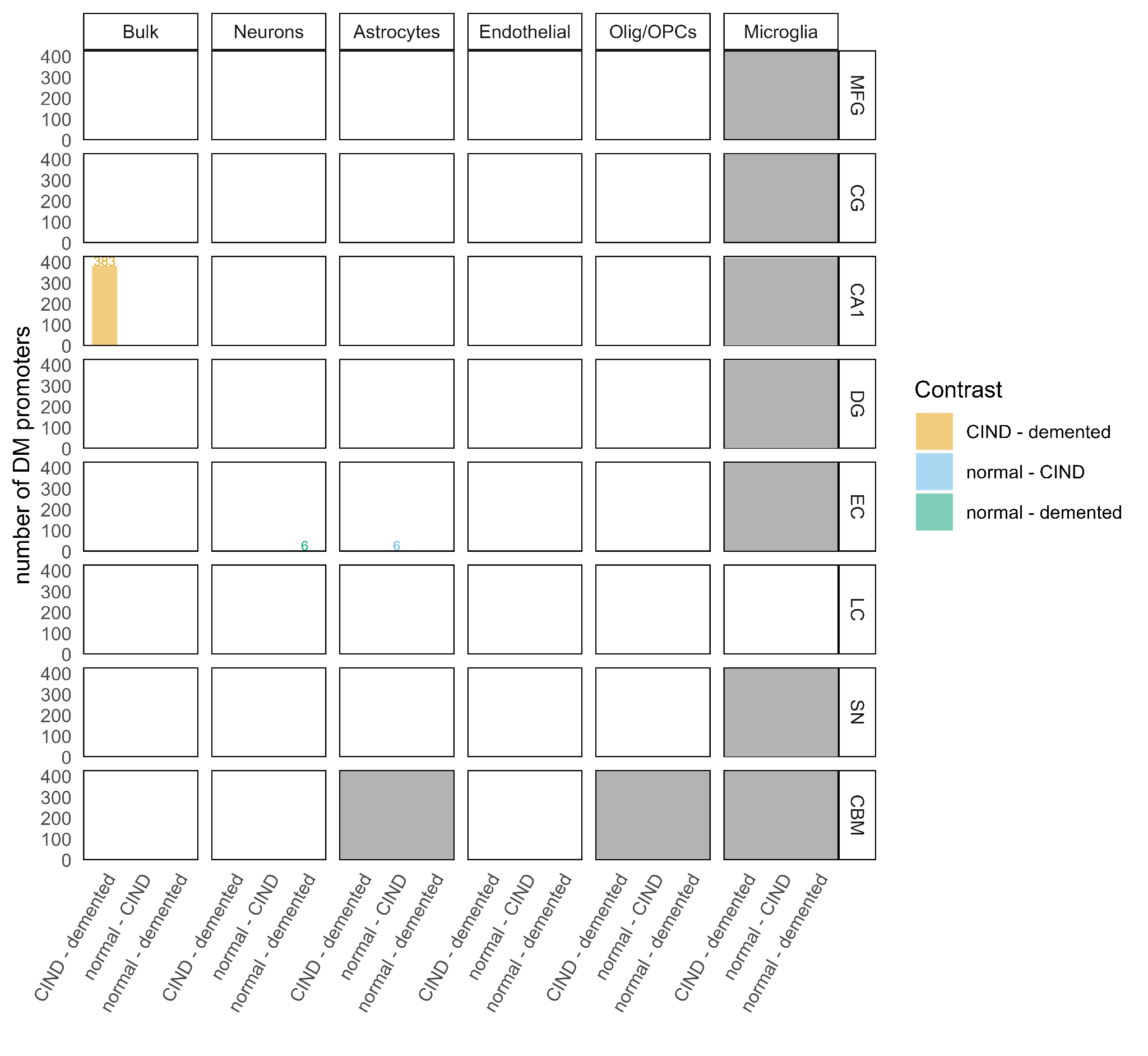


### Supplementary Figure 12: Overview of brain region and cell type specific differentially methylated promoter regions of protein coding genes across clinical diagnosis of dementia

Barplots display the number of significant (FDR < 0.05) differentially methylated promoter regions of protein coding genes. The plot is split by cell type as columns and brain regions as rows. Color coding of the bars reflects the different categorical comparisons of clinical dementia diagnosis: normal, cognitive impaired not demented (CIND), demented. Comparisons highlighted in gray were not analyzed due to weak cell type specific methylation signals within the given brain region (see Methods). Cell type specific differential methylation was detected in neurons (normal vs demented) and astrocytes (normal vs CIND) of the entorhinal cortex (EC). Abbreviations: *Olig/OPCs* Oligodendrocytes/Oligodendrocyte Precursor Cells, *MFG* Middle Frontal Gyrus, *CG* Cingulate Gyrus, *DG* Dentate Gyrus, *EC* Entorhinal cortex, *SN* Substantia nigra, *LC*  Locus coeruleus, *CBM* Cerebellar cortex.
